# CRISPR/Cas9: a molecular Swiss army knife for simultaneous introduction of multiple genetic modifications in *Saccharomyces cerevisiae*

**DOI:** 10.1093/femsyr/fov004

**Published:** 2015-03-17

**Authors:** Robert Mans, Harmen M. van Rossum, Melanie Wijsman, Antoon Backx, Niels G.A. Kuijpers, Marcel van den Broek, Pascale Daran-Lapujade, Jack T. Pronk, Antonius J.A. van Maris, Jean-Marc G. Daran

**Affiliations:** Department of Biotechnology, Delft University of Technology, Julianalaan 67, 2628 BC Delft, The Netherlands

**Keywords:** CRISPR/Cas9, *S. cerevisiae*, gRNA, genetic modification, webtool, plasmid

## Abstract

A variety of techniques for strain engineering in *Saccharomyces cerevisiae* have recently been developed. However, especially when multiple genetic manipulations are required, strain construction is still a time-consuming process. This study describes new CRISPR/Cas9-based approaches for easy, fast strain construction in yeast and explores their potential for simultaneous introduction of multiple genetic modifications. An open-source tool (http://yeastriction.tnw.tudelft.nl) is presented for identification of suitable Cas9 target sites in *S. cerevisiae* strains. A transformation strategy, using *in vivo* assembly of a guideRNA plasmid and subsequent genetic modification, was successfully implemented with high accuracies. An alternative strategy, using *in vitro* assembled plasmids containing two gRNAs, was used to simultaneously introduce up to six genetic modifications in a single transformation step with high efficiencies. Where previous studies mainly focused on the use of CRISPR/Cas9 for gene inactivation, we demonstrate the versatility of CRISPR/Cas9-based engineering of yeast by achieving simultaneous integration of a multigene construct combined with gene deletion and the simultaneous introduction of two single-nucleotide mutations at different loci. Sets of standardized plasmids, as well as the web-based Yeastriction target-sequence identifier and primer-design tool, are made available to the yeast research community to facilitate fast, standardized and efficient application of the CRISPR/Cas9 system.

## Abstract (precis)

CRISPR/Cas9 like a Swiss army knife enables molecular biologists to quickly introduce simultaneous multiple and diverse genetic modifications in baker's yeast *Saccharomyces cerevisiae*.

## Graphical abstract

**Graphical Abstract Figure:**
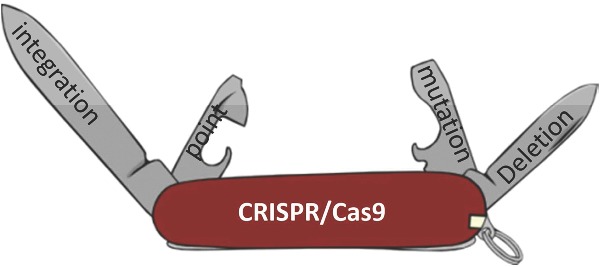
CRISPR/Cas9 like a Swiss army knife enables molecular biologists to quickly introduce simultaneous multiple and diverse genetic modifications in baker's yeast *Saccharomyces cerevisiae*.

## INTRODUCTION

For decades*, Saccharomyces cerevisiae* has been successfully used as a model organism to decipher biological processes in higher eukaryotes (Botstein and Fink 2011) and as a popular metabolic engineering platform (Nielsen *et al.* 2013). Expression and optimization of heterologous product pathways in *S. cerevisiae* (see e.g. Paddon *et al.* 2013; Beekwilder *et al.* 2014) requires introduction of multiple (successive) genetic modifications, including integration of product pathway genes at multiple genetic loci and rewiring central metabolism by modifying properties of specific metabolic reactions (e.g. via gene deletion, changing regulatory properties or replacement of native genes by heterologous counterparts) (Wieczorke *et al.* 1999; Ro *et al.* 2006). Introduction of the required genetic modifications has so far remained a time-consuming and labour-intensive process, as each individual alteration requires a cycle of transformation, selection and confirmation. Furthermore, since each modification is accompanied by the integration of a selection marker gene, the maximum number of sequential modifications may be limited by selection marker availability. This limitation stimulated extensive research into the identification of novel genetic markers for *S. cerevisiae* (Chee and Haase 2012; Solis-Escalante *et al.* 2013; Siewers 2014). Additionally, multiple strategies for the recycling of genetic markers have been developed, such as homologous recombination (HR)-mediated counter selection (gene ‘loop-out’) and use of recombinases such as the Cre/*loxP* or 2 μm-plasmid-based Flp/FRT methods (Güldener *et al.* 1996; Storici, Coglievina and Bruschi 1999; Hegemann and Heick 2011). These recombinase-based methods leave a copy of a repeat sequence (e.g. *loxP* or FRT site) in the genome, which leads to genome instability after multiple, repeated rounds of marker recovery (Delneri *et al.* 2000; Solis-Escalante *et al.* 2014b). ‘Scarless’ removal of counter-selectable markers has been made possible via the *delitto perfetto* method (Storici, Lewis and Resnick 2001), while a recently reported marker-recovery method based on generation of I-SceI-induced double-stranded breaks even allows simultaneous, seamless removal of multiple markers (Solis-Escalante *et al.* 2014a). While these methods largely eliminate limitations by marker-gene availability, substitution of target genes by marker cassettes remained a time-consuming process, due to the absence of robust methods for simultaneous introduction of multiple genetic modifications in a single transformation step. Alternative methods such as meganucleases, zinc finger nucleases (ZFNs) (Urnov *et al.* 2010; Carroll 2011) and transcription activator-like effector nucleases (TALENs) (Christian *et al.* 2010; Miller *et al.* 2011; Mussolino *et al.* 2011) utilize double-stranded DNA breaks (DSBs) for site-directed genome editing. Due to the lethal nature of DSBs in yeast, these methods could theoretically be used for marker-free modifications. However, for each genetic modification, a new ZFN or TALEN protein has to be designed and generated.

Bacteria have developed several systems to degrade foreign DNA. Very quickly after their discovery, restriction enzymes became the ‘workhorses of molecular biology’ (reviewed by Roberts 2005). Another prokaryotic immune mechanism, consisting of Clustered Regularly Interspaced Short Palindromic Repeats (CRISPR) and CRISPR-associated (Cas) systems, was discovered in 2007 (Barrangou *et al.* 2007; Brouns *et al.* 2008; Marraffini and Sontheimer 2008). To function *in vivo*, the type-II bacterial CRISPR system of *Streptococcus pyogenes* requires the Cas9 nuclease and the RNA complex that guides it to a specific sequence of the (foreign) DNA. This RNA complex generally consists of two RNA molecules: the CRISPR RNA (crRNA) and the trans-activating CRISPR RNA (tracrRNA). The crRNA contains the 20–30 base pairs (bp) target sequence and a sequence that binds to the tracrRNA, resulting in a duplex RNA complex, recognized by the Cas9 nuclease. When directly after the target sequence a proper protospacer adjacent motif (PAM) is present (in case of the *S. pyogenes* Cas9 this sequence is NGG), Cas9 will bind to and restrict the target sequence of the (invading) DNA (Deltcheva *et al.* 2011). A unique feature of this system is the RNA dependence for targeting of the nuclease Cas9, which makes selective targeting of any locus for the introduction of DSBs possible. Since its discovery, Cas9-based systems have been used for the construction of (multiplexed) genetic modifications in a variety of organisms, including human pluripotent stem cells (González *et al.* 2014), zebrafish (Hwang *et al.* 2013), plants (Feng *et al.* 2013), flies (Gratz *et al.* 2013) and mice (Wang *et al.* 2013; for a more extensive list, see Hsu, Lander and Zhang 2014).

In 2013, DiCarlo *et al.* (2013b) employed the CRISPR/Cas9 system for the introduction of DSBs in *S. cerevisiae*. In a strain expressing a plasmid-borne *cas9* gene from *S. pyogenes*, a second plasmid was introduced, containing the *SNR52* promoter followed by a sequence encoding for a chimeric crRNA-tracrRNA, or guide-RNA (gRNA) with a 20 bp targeting sequence for *CAN1*. The gRNA was recognized by the Cas9 protein, resulting in a double-strand break at the *CAN1* locus. Subsequently, this otherwise lethal break was repaired by the yeast HR machinery, using a co-transformed repair fragment that bridged the flanking regions of the break. Since the repair fragment was designed to introduce a premature stop codon, introduction and repair of the DSB resulted in colonies that were resistant to canavanine. It has recently been shown that the CRISPR/Cas9 system can be used for making up to three simultaneous gene deletions in yeast (Bao *et al.* 2014).

The goal of the present study is to explore the use of CRISPR/Cas9 for standardized (multiplexed) construction of gene deletions, multipathway integrations and site-directed single-nucleotide mutagenesis. To this end, we present a web-based CRISPR tool to facilitate selection of suitable targets and to design the primers necessary for construction of plasmids that express specific gRNAs. Furthermore, we report on the construction of standardized plasmids for expression of one or two gRNAs and explore their use for multiplexed gene deletions, both alone and in combination with multigene chromosomal integrations and/or with the introduction of single-nucleotide changes.

## MATERIALS AND METHODS

### Strains, growth conditions and storage

The *S. cerevisiae* strains used in this study (Table 1) share the CEN.PK genetic background (Entian and Kötter 2007; Nijkamp *et al.* 2012). Shake flask cultures were grown at 30 °C in 500 mL flasks containing 100 mL synthetic medium (SM) (Verduyn *et al.* 1992) with 20 g·L^−1^ glucose in an Innova incubator shaker (New Brunswick Scientific, Edison, NJ, USA) set at 200 rpm. When required, auxotrophic requirements were complemented via addition of 150 mg·L^−1^ uracil, 100 mg·L^−1^ histidine, 500 mg·L^−1^ leucine, 75 mg·L^−1^ tryptophan (Pronk, 2002) or by growth in YP medium (demineralized water, 10 g·L^−1^ Bacto yeast extract, 20 g·L^−1^ Bacto peptone). As a carbon source, 20 g·L^−1^ glucose was used. Frozen stocks were prepared by addition of glycerol (30% v/v) to exponentially growing shake-flask cultures of *S. cerevisiae* and overnight cultures of *Escherichia coli* and stored aseptically in 1 mL aliquots at –80 °C.

**Table 1.** *Saccharomyces cerevisiae* strains used in this study.

Name (Accession no.)	Relevant genotype	Parental strain	Origin
CEN.PK113-7D	*MAT*a *URA3 TRP1 LEU2 HIS3*		P. Kötter
CEN.PK113-5D	*MAT*a *ura3-52 TRP1 LEU2 HIS3*		P. Kötter
CEN.PK122	*MAT*a/*MATα URA3/URA3 TRP1/TRP1 LEU2/LEU2 HIS3/HIS3*		P. Kötter
CEN.PK2-1C	*MAT*a *ura3-52 trp1-289 leu2-3,112 his3Δ*		P. Kötter
CEN.PK115	*MAT*a/*MATα ura3-52/ura3-52 TRP1/TRP1 LEU2/LEU2 HIS3/HIS3*		P. Kötter
IMX585 (Y40592)	*MAT*a *can1Δ::cas9-natNT2 URA3 TRP1 LEU2 HIS3*	CEN.PK113-7D	This study
IMX581 (Y40593)	*MAT*a *ura3-52 can1Δ::cas9-natNT2 TRP1 LEU2 HIS3*	CEN.PK113-5D	This study
IMX664 (Y40594)	*MAT*a/*MATα CAN1/can1Δ::cas9-natNT2 URA3/URA3 TRP1/TRP1 LEU2/LEU2 HIS3/HIS3*	CEN.PK122	This study
IMX672 (Y40595)	*MAT*a *ura3-52 trp1-289 leu2-3,112 his3Δ can1Δ::cas9-natNT2*	CEN.PK2-1C	This study
IMX673 (Y40596)	*MAT*a/*MATα ura3-52/ ura3-52 CAN1/can1Δ::cas9-natNT2 TRP1/TRP1 LEU2/LEU2 HIS3/HIS3*	CEN.PK115	This study
IMX711	*MAT*a *ura3-52 trp1-289 leu2-3,112 his3Δ can1Δ::cas9-natNT2 mch1Δ* pMEL10*-*gRNA-*MCH1*	IMX672	This study
IMX712	*MAT*a *ura3-52 trp1-289 leu2-3,112 his3Δ can1Δ::cas9-natNT2 mch2Δ* pMEL10*-*gRNA-*MCH2*	IMX672	This study
IMX713	*MAT*a *ura3-52 trp1-289 leu2-3,112 his3Δ can1Δ::cas9-natNT2 mch5Δ* pMEL10*-*gRNA-*MCH5*	IMX672	This study
IMX714	*MAT*a *ura3-52 trp1-289 leu2-3,112 his3Δ can1Δ::cas9-natNT2 mch1Δ mch5Δ* pMEL10*-*gRNA-*MCH1* pMEL10*-*gRNA-*MCH5*	IMX672	This study
IMX715	*MAT*a *ura3-52 trp1-289 leu2-3,112 his3Δ can1Δ::cas9-natNT2 itr1Δ pdr12Δ* pUDR005	IMX672	This study
IMX716	*MAT*a *ura3-52 trp1-289 leu2-3,112 his3Δ can1Δ::cas9-natNT2 mch1Δ mch2Δ itr1Δ pdr12Δ* pUDR002 pUDR005	IMX672	This study
IMX717	*MAT*a *ura3-52 trp1-289 leu2-3,112 his3Δ can1Δ::cas9-natNT2 mch1Δ mch2Δ mch5Δ aqy1Δ itr1Δ pdr12Δ* pUDR002 pUDR004 pUDR005	IMX672	This study
IMX718	*MAT*a *ura3-52 trp1-289 leu2-3,112 his3Δ can1Δ::cas9-natNT2 GET4^G315C^ NAT1^C1139G^* pUDR020	IMX672	This study
IMX719	*MAT*a *can1Δ::cas9-natNT2 URA3 TRP1 LEU2 HIS3 acs1Δ acs2Δ:*:*(pADH1-aceF-tPGI1 pPGI1-lplA2-tPYK1 pPGK1-lplA-tPMA1 pTDH3-pdhB-tCYC1 pTEF1-lpd-tADH1 pTPI1-pdhA-tTEF1)*	IMX585	This study

Strains with an accession number have been deposited at Euroscarf (http://web.uni-frankfurt.de/fb15/mikro/euroscarf/).

**Table 2.** Plasmids used in this study.

Name (Accession no.)	Relevant characteristics	Origin
pUG6	Template for A-*kanMX*-B† cassette	(Gueldener *et al.* 2002)
pUG72	Template for A-*KlURA3*-B cassette	(Gueldener *et al.* 2002)
pUG73	Template for A-*KlLEU2*-B cassette	(Gueldener *et al.* 2002)
pUG-hphNT1	Template for A-*hphNT1*-B cassette	(de Kok *et al.* 2011)
pUG-natNT2	Template for A-*natNT2*-B cassette	(de Kok *et al.* 2012)
pUG-amdSYM	Template for A-*amdSYM*-B cassette	(Solis-Escalante *et al.* 2013)
pRS423	Template for A-*HIS3-*B cassette	(Christianson *et al.* 1992)
pRS424	Template for A-*TRP1*-B cassette	(Christianson *et al.* 1992)
p414-TEF1p-Cas9-CYC1t	*CEN6/ARS4* ampR *TRP1 pTEF1-cas9-tCYC1*	(DiCarlo *et al.* 2013a)
p426-SNR52p-gRNA.CAN1.Y-SUP4t	2μm ampR *URA3* gRNA-*CAN1*.Y	(DiCarlo *et al.* 2013a)
pUD192	pUC57 + *URA3*	(Kozak *et al.* 2014a)
pUD194	pUC57 + 2μm	(Kozak *et al.* 2014a)
pUD195	pUC57 + pMB1 + ampR	(Kozak *et al.* 2014a)
pUD301	pUC57 + *pTPI1-pdhA E. faecalis-tTEF1*	(Kozak *et al.* 2014a)
pUD302	pUC57 + *pTDH3-pdhB E. faecalis -tCYC1*	(Kozak *et al.* 2014a)
pUD303	pUC57 + *pADH1-aceF E. faecalis -tPGI1*	(Kozak *et al.* 2014a)
pUD304	pUC57 + *pTEF1-lpd E. faecalis -tADH1*	(Kozak *et al.* 2014a)
pUD305	pUC57 + *pPGK1-lplA E. faecalis -tPMA1*	(Kozak *et al.* 2014a)
pUD306	pUC57 + *pPGI1-lplA2 E. faecalis -tPYK1*	(Kozak *et al.* 2014a)
pUDE330	2μm ampR *URA3* gRNA-*CAN1*.Y [2x]	This study
pMEL10 (P30779)	2μm ampR *KlURA3* gRNA-*CAN1*.Y	This study
pMEL11 (P30780)	2μm ampR *amdSYM* gRNA-*CAN1*.Y	This study
pMEL12 (P30781)	2μm ampR *hphNT1* gRNA-*CAN1*.Y	This study
pMEL13 (P30782)	2μm ampR *kanMX* gRNA-*CAN1*.Y	This study
pMEL14 (P30783)	2μm ampR *KlLEU2* gRNA-*CAN1*.Y	This study
pMEL15 (P30784)	2μm ampR *natNT2* gRNA-*CAN1*.Y	This study
pMEL16 (P30785)	2μm ampR *HIS3* gRNA-*CAN1*.Y	This study
pMEL17 (P30786)	2μm ampR *TRP1* gRNA-*CAN1*.Y	This study
pROS10 (P30787)	2μm ampR *URA3* gRNA-*CAN1*.Y gRNA*-ADE2*.Y	This study
pROS11 (P30788)	2μm ampR *amdSYM* gRNA*-CAN1*.Y gRNA*-ADE2*.Y	This study
pROS12 (P30789)	2μm ampR *hphNT1* gRNA*-CAN1*.Y gRNA*-ADE2*.Y	This study
pROS13 (P30790)	2μm ampR *kanMX* gRNA-*CAN1*.Y gRNA*-ADE2*.Y	This study
pROS14 (P30791)	2μm ampR *KlLEU2* gRNA*-CAN1*.Y gRNA*-ADE2*.Y	This study
pROS15 (P30792)	2μm ampR *natNT2* gRNA*-CAN1*.Y gRNA*-ADE2*.Y	This study
pROS16 (P30793)	2μm ampR *HIS3* gRNA*-CAN1*.Y gRNA*-ADE2*.Y	This study
pROS17 (P30794)	2μm ampR *TRP1* gRNA*-CAN1*.Y gRNA*-ADE2*.Y	This study
pUDR002	2μm ampR *TRP1* gRNA*-MCH1* gRNA*-MCH2*	This study
pUDR004	2μm ampR *HIS3* gRNA*-MCH5* gRNA*-AQY1*	This study
pUDR005	2μm ampR *URA3* gRNA*-ITR1* gRNA*-PDR12*	This study
pUDR020	2μm ampR *URA3* gRNA*-NAT1* gRNA*-GET4*	This study
pUDR022	2μm ampR *kanMX* gRNA*-ACS1* gRNA*-ACS2*	This study

^†^A and B refer to 60 bp tags that are incorporated via PCR, enabling homologous recombination.

Plasmid with an accession number have been deposited at Euroscarf (http://web.uni-frankfurt.de/fb15/mikro/euroscarf/).

### Plasmid construction

#### Construction of the single gRNA plasmid series (pMEL10–pMEL17)

The single gRNA plasmids (pMEL10–pMEL17) were constructed via Gibson assembly (New England Biolabs, Beverly, MA, USA) of a marker cassette with one fragment containing both the gRNA *CAN1*.Y (DiCarlo *et al.* 2013b) and the 2 μm replication sequence. This fragment was obtained by PCR from plasmid p426-SNR52p-gRNA.CAN1.Y-SUP4t, using primers 6845 & 6846 (Table S1, Supplementary data). The various marker cassettes were PCR amplified from plasmid templates pUG72, pUG-amdSYM, pUG-hphNT1, pUG6, pUG73 and pUG-natNT2 with primers 3093 & 3096 resulting in the *KlURA3, amdSYM, hphNT1, kanMX, KlLEU2* and *natNT2* cassettes, respectively. The *HIS3* and *TRP1* cassettes were obtained by PCR with primers 6847 & 6848 on plasmid templates pRS423 and pRS424. Assembly of the single gRNA plasmids was done by combining the appropriate marker cassette with the backbone containing the gRNA *CAN1*.Y and 2 μm sequences in a Gibson assembly reaction, following the manufacturer's recommendations. For each single gRNA plasmid (pMEL10–pMEL17), an *E. coli* clone containing the correctly assembled plasmid (confirmed by restriction analysis) was selected, stocked and deposited at EUROSCARF (http://web.uni-frankfurt.de/fb15/mikro/euroscarf/).

#### Construction of the double gRNA plasmid series (pROS10 – pROS17)

To construct the double gRNA plasmids (pROS10–pROS17), an intermediate plasmid was first constructed, carrying two gRNA cassettes that both targeted *CAN1*.Y (DiCarlo *et al.* 2013b). This intermediate plasmid was assembled out of four different overlapping fragments: the two gRNA cassettes overlapping with each other in the 2 μm replicon, one *URA3* marker cassette and a cassette containing all sequences for amplification in *E. coli*. The gRNA cassettes were obtained in a two-step PCR approach. First, a 2 μm fragment was obtained from pUD194 (Table 2) with primers 3289 & 4692 and two different gRNA cassettes were PCR amplified from p426-SNR52p-gRNA.CAN1.Y-SUP4t with primers 5972 & 5976 for the first cassette and 5977 & 5973 for the second cassette. Each gRNA cassette was separately pooled with the 2 μm fragment and in a second PCR reaction, the gRNA cassettes were extended with either the 5^′^ or 3^′^ halve of the 2 μm fragment, resulting in two different gRNA cassettes, overlapping in the 2 μm sequence, by using primer pair 5975 & 4068 for the first and 5974 & 3841 for the second fragment. The marker fragment containing *URA3* was obtained from pUD192 with primers 3847 & 3276 (Table 2) and the fragment containing all sequences for amplification in *E. coli* was PCR amplified from pUD195 (Table 2) with primers 3274 & 3275. Using Gibson assembly, the four overlapping fragments were assembled into the intermediate plasmid pUDE330. To obtain pROS10, pUDE330 was linearized by PCR amplification of the backbone, excluding the gRNA fragments, and co-transformed with two gRNA cassettes for *in vivo* assembly by HR in yeast (Kuijpers *et al.* 2013b). For linearizing the backbone, a single primer was used (5793) and the gRNA fragments were obtained by PCR from pUDE330 with primers 6008 & 5975 and 6007 & 5974. The plasmid was extracted from yeast and transformed into *E. coli* for storage and plasmid propagation. The other double gRNA plasmids were assembled by the Gibson assembly method with a marker cassette and the pROS10 plasmid backbone fragment. This backbone fragment was obtained by linearization of pROS10 with restriction enzymes *Pvu*II and *Not*I. The various marker cassettes were PCR amplified from plasmid templates pUG-amdSYM, pUG-hphNT1, pUG6, pUG73 and pUG-natNT2 with primers 3093 & 3096 resulting in the *amdSYM, hphNT1, kanMX, KlLEU2* and *natNT2* cassettes, respectively. The *HIS3* and *TRP1* cassettes were obtained by PCR with primers 6847 & 6848 on plasmid templates pRS423 and pRS424. Combining the appropriate marker cassette with the pROS10 backbone fragment in a Gibson assembly reaction, following the manufacturer's recommendations, resulted in pROS11–pROS17. For each of these double gRNA plasmids, an *E. coli* clone containing the correctly assembled plasmid was selected, stocked and deposited at EUROSCARF (http://web.uni-frankfurt.de/fb15/mikro/euroscarf/).

### Strain construction

*Saccharomyces cerevisiae* strains were transformed according to Gietz and Woods (2002). Mutants were selected on solid YP medium (demineralized water, 10 g·L^−1^ Bacto yeast extract, 20 g·L^−1^ Bacto peptone, 2% (w/v) agar), supplemented with 200 mg·L^−1^ G418, 200 mg·L^−1^ hygromycin B or 100 mg·L^−1^ nourseothricin (for dominant markers) or on SM supplemented with appropriate auxotrophic requirements (Verduyn *et al.* 1992). In all cases, gene deletions and integrations were confirmed by colony PCR on randomly picked colonies, using the diagnostic primers listed in Table S1 (Supplementary data). Integration of *cas9* into the genome was achieved via assembly and integration of two cassettes containing *cas9* and the *natNT2* marker into the *CAN1* locus. The *cas9* cassette was obtained by PCR from p414-TEF1p-cas9-CYC1t (DiCarlo *et al.* 2013b), using primers 2873 & 4653. The *natNT2* cassette was PCR amplified from pUG-natNT2 with primers 3093 & 5542. 2.5 μg *cas9* and 800 ng *natNT2* cassette were pooled and used for each transformation. Correct integration was verified by colony PCR (Supplementary data) using the primers given in Table S1 (Supplementary data), the resulting strains have been deposited at EUROSCARF. IMX719 was constructed by co-transformation of pUDR022 (see below) with genes required for functional *Enterococcus faecalis* PDH expression (Kozak *et al.* 2014b). The gene cassettes were obtained by PCR using plasmids pUD301–pUD306 as template (Table 2) with the primers indicated in Table S1 (Supplementary data) and the *ACS1* dsDNA repair fragment, obtained by annealing two complementary single-stranded oligos (6422 & 6423). After confirmation of the relevant genotype (Fig. 4B), the pUDR022 plasmid was removed as explained in Supplementary data.

**Figure 1.**
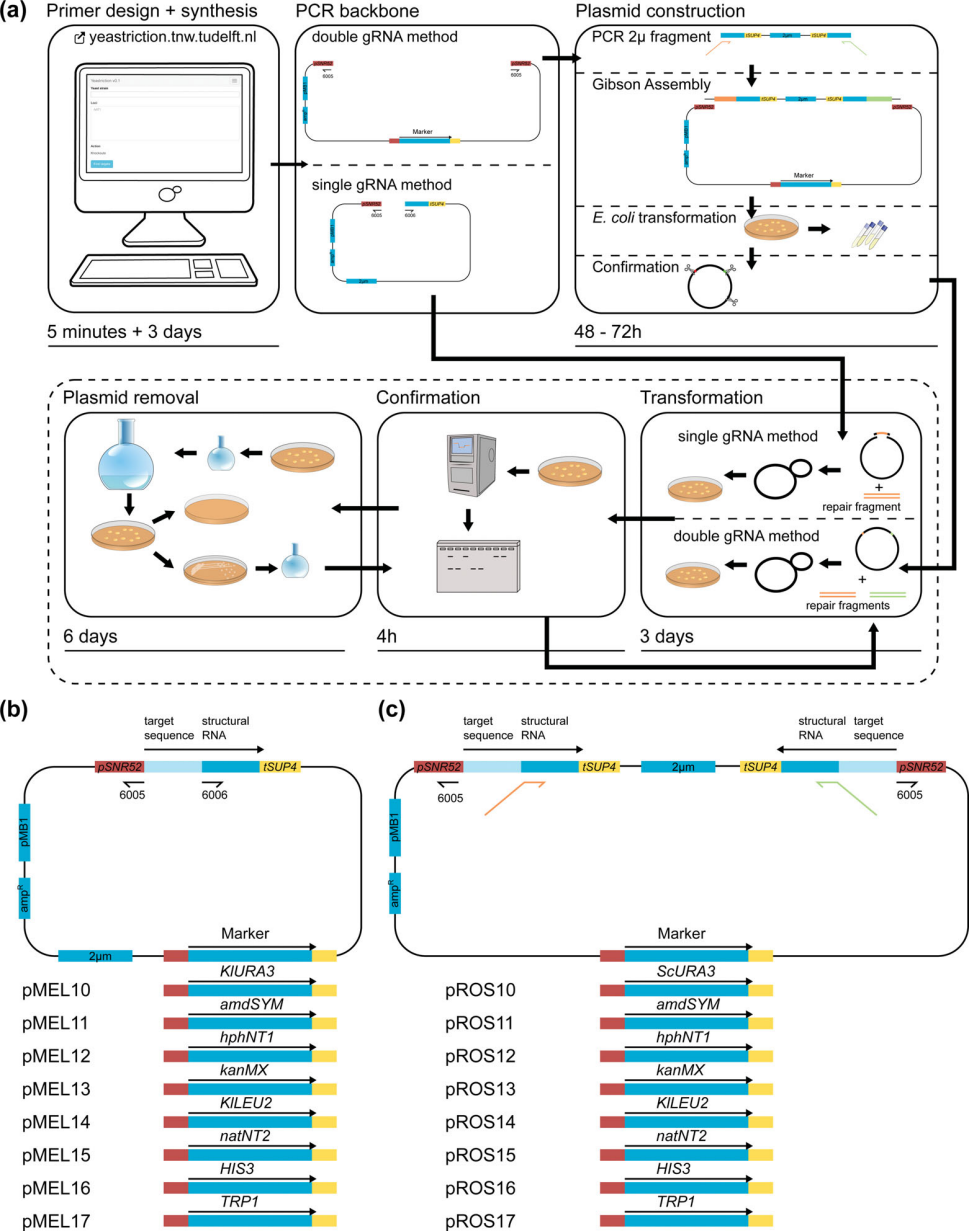
Workflow for CRISPR/Cas9 modification of *S. cerevisiae* genome using single and double gRNA plasmid series (pMEL10–pMEL17 and pROS10–pROS17, respectively). **(a)** All oligonucleotides, required for targeting the gene(s) of interest (GOI), can be automatically designed with the Yeastriction webtool (http://yeastriction.tnw.tudelft.nl). For the single gRNA method, the tool designs complementary oligonucleotides that can be annealed to form (i) a double-stranded repair fragment and (ii) a double-stranded insert which contains the target sequence for the GOI. For expression of the gRNA, a plasmid backbone containing the genetic marker of choice is amplified from a single gRNA plasmid (pMEL10–pMEL17). Gene deletion is achieved via co-transformation of the plasmid backbone, the dsDNA insert (containing the gRNA target sequence, flanked by sequences identical to both sides of the linearized plasmid backbone) and the repair fragment. For the double gRNA method, Yeastriction designs two sets of oligonucleotides, the first oligonucleotide binds to the 2 μm fragment and has a tail containing the desired target sequence for the GOI and a sequence identical to both sides of a linearized double gRNA plasmid backbone (pROS10–pROS17), the second set of oligonucleotides can be annealed to form the dsDNA repair fragment(s). To construct a gRNA plasmid with two target sequences, first a double gRNA plasmid backbone with the appropriate marker (pROS10–pROS17) is amplified by PCR, excluding the 2 μm fragment. Then, the 2 μm fragment is PCR amplified using the primers harbouring the targets for the GOIs and sequences identical to the plasmid backbone. The final plasmid is then constructed *in vitro* using the plasmid backbone and the 2 μm fragment, e.g. with the Gibson assembly method. After confirmation of correct plasmid assembly using restriction analysis or PCR, the resulting plasmid is transformed to yeast, together with the appropriate 120 bp dsDNA repair fragment(s). After transformation, the desired genetic modification(s) are checked by PCR, Southern blot analysis or sequencing. Subsequently, the strain can be modified again in a new round of transformation (preferably using plasmids with other markers). Before physiological analysis, the gRNA plasmid(s) are preferably removed. This can be done by growing the strains in liquid media without selection pressure or, if possible, by counter-selection pressure (with 5-fluoroorotic acid, fluoroacetamide or 5-fluoroanthranilic acid for pMEL10+pROS10 plasmids, pMEL11+pROS11 plasmids and pMEL17+pROS17 plasmids, respectively). After confirming plasmid removal by restreaking the same colony on selective and non-selective medium and/or PCR analysis, the resulting strain is re-grown in liquid medium and stored at –80 °C. **(b)** Architecture of the single gRNA plasmid series (pMEL10–pMEL17). The primers used for PCR amplification of the plasmid backbone are indicated by black arrows. **(c)** Architecture of the double gRNA plasmid series (pROS10–pROS17) with two gRNA cassettes. The plasmid backbone can be PCR amplified with a single primer (indicated with a black arrow). The 2 μm fragment is amplified with primers designed using Yeastriction (indicated in orange and light green coloured arrows).

**Figure 2.**
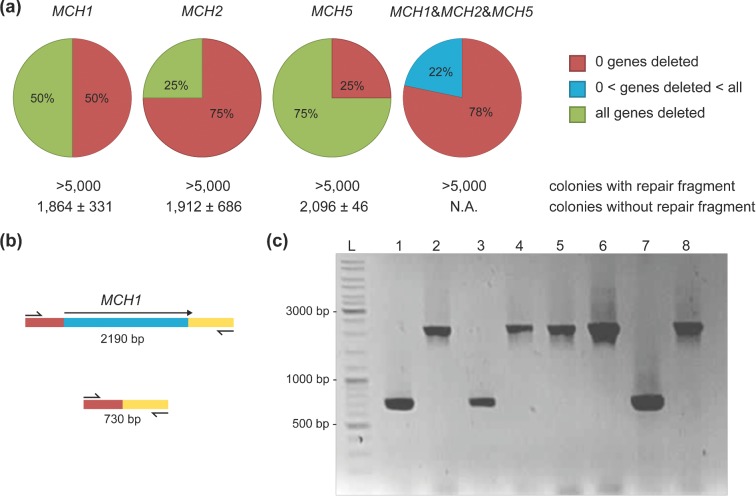
Efficiency of gene deletion obtained after transformation with a single gRNA plasmid. **(a)** Quantification of the number of colonies and corresponding gene deletion efficiencies, obtained after transformation of *S. cerevisiae* IMX672 (*ura3–52 trp1–289 leu2–3,112 his3*Δ, *can1*Δ*::cas9*) with 100 ng pMEL10 backbone, 300 ng of gRNA insert DNA and 2 μg of the corresponding 120 bp dsDNA repair fragment. The transformation targeting *MCH1, MCH2* and *MCH5* simultaneously was performed using 300 ng of each insert and 2 μg of each repair fragment. For the transformations with repair fragments, the exact number of transformants could not be determined, but exceeded 5000 colonies per plate. The data represent average and standard deviation of transformants of three independent transformation experiments. The estimated total number of colonies carrying gene deletions was based on colony PCR results of 24 randomly picked colonies. In red: percentage of colonies without gene deletions, in blue: percentage of colonies containing one or two but not all deletions, green: percentage of colonies containing all desired gene deletions. No transformants with all three genes deleted were identified. **(b)** Diagnostic primers were designed outside of the target ORFs to differentiate between successful and non-successful colonies via PCR. In this colony PCR example, primers 6862 & 6863 were used to amplify the *MCH1* locus. **(c)** Example of a diagnostic gel from the transformation targeting the *MCH1* locus. The first lane (L) contains the GeneRuler DNA Ladder Mix. Lane 1–8 show the PCR results of eight randomly picked colonies. Successful deletion of *MCH1* results in a PCR fragment with a length of 729 bp (lane 1, 3 and 7), when *MCH1* is still present a band is observed at 2190 bp (lane 2, 4, 5, 6 and 8).

**Figure 3.**
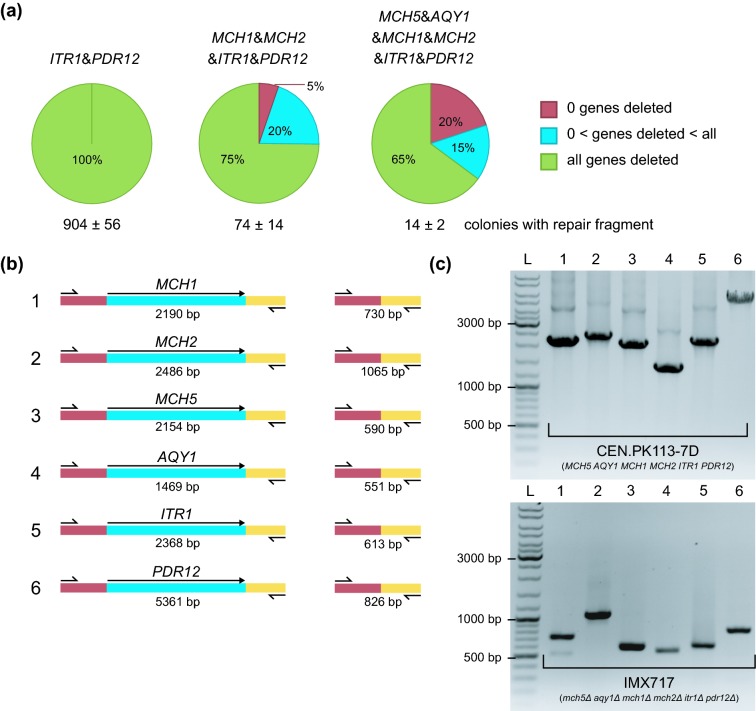
Efficiency of gene deletion obtained after transformation with double gRNA plasmids. **(a)** Quantification of the number of colonies and corresponding gene deletion efficiencies after transformation of *S. cerevisiae* IMX672 (*ura3–52 trp1–289 leu2–3,112 his3*Δ, *can1*Δ*::cas9*) with 2 μg of various double gRNA plasmids with 1 μg of the appropriate repair fragment(s). When multiple plasmids were transformed simultaneously, 2 μg of each plasmid was added. The data represent average and standard deviation of transformants of three independent transformation experiments. In red: percentage of colonies containing no gene deletions, in blue: percentage of colonies containing some but not all targeted gene deletions (1, 1–3 and 1–5 respectively), in green: percentage of colonies containing all targeted simultaneous gene deletions (2, 4 and 6 respectively). **(b)** Diagnostic primers were designed outside of the target ORFs to differentiate between successful and non-successful colonies via PCR. In this colony PCR example, primers 6862 & 6863, 6864 & 6865, 6866 & 6867, 6868 & 6870, 6869 & 6870 and 253 & 3998 were used to amplify the *MCH1, MCH2, MCH5, AQY1, ITR1* and *PDR12* loci, respectively. The expected sizes of the PCR products obtained when the gene is present (left) or deleted (right) are indicated. **(c)** Example of a diagnostic gel from the transformation introducing six simultaneous gene deletions, resulting in IMX717. The first lane (L) contains the GeneRuler DNA Ladder Mix. Lane 1–6 show the PCR results of the reference strain CEN.PK113–7D (top) and a randomly picked colony of IMX717 (bottom) with primers 6862 & 6863 for *MCH1* (lane 1), 6864 & 6865 for *MCH2* (lane 2), 6866 & 6867 for *MCH5* (lane 3), 6868 & 5593 for *AQY1* (lane 4), 6869 & 6870 *ITR1* (lane 5) and 253 & 3998 for *PDR12* (lane 6).

**Figure 4.**
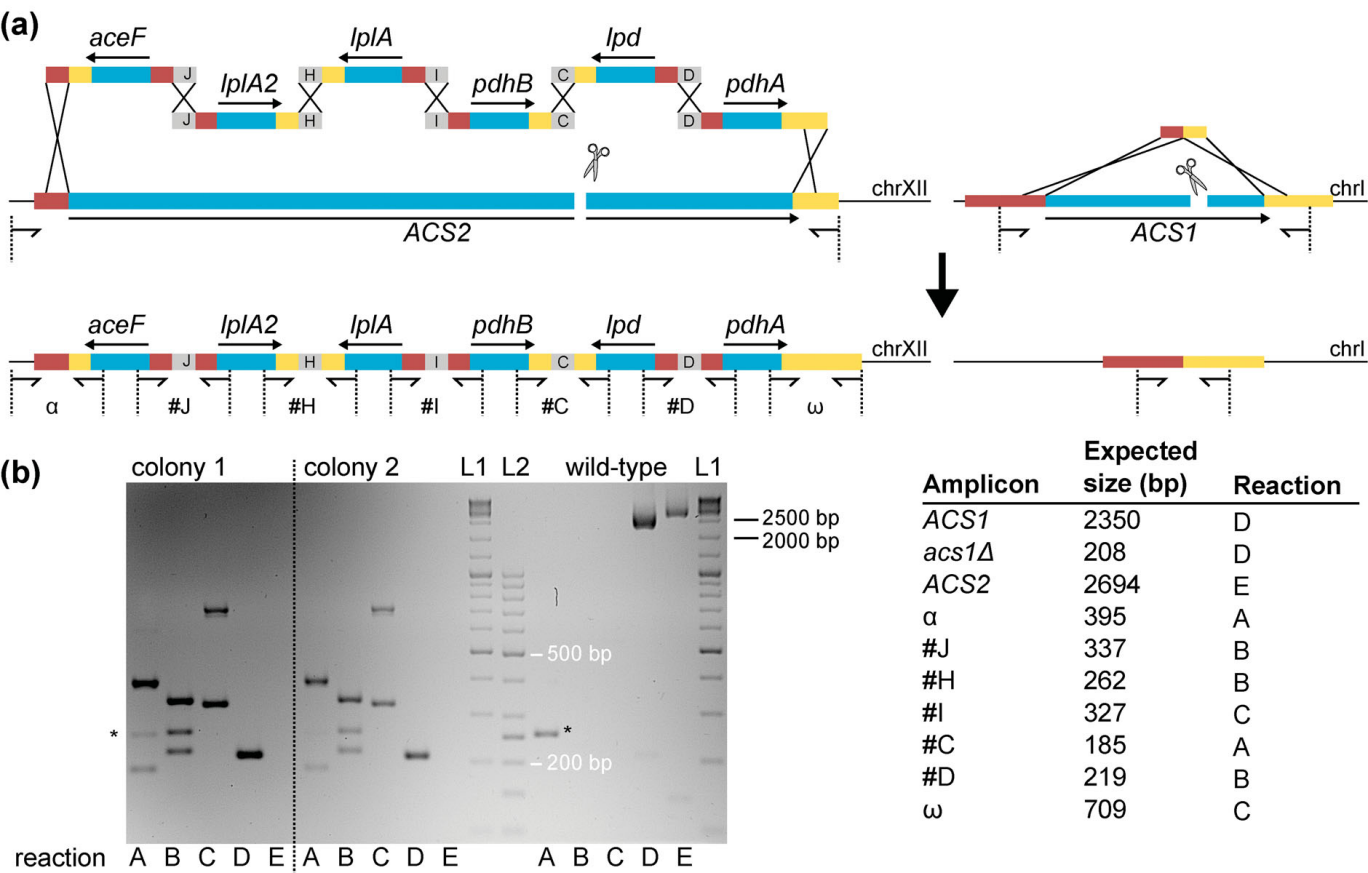
Multiplexing CRISPR/Cas9 in *S. cerevisiae*. (**a**) Chromosomal integration of the six genes required for expression of a functional *E. faecalis* pyruvate dehydrogenase complex in the yeast cytosol. All six genes are flanked by 60 bp sequences enabling HR (indicated with black crosses). The first and the last fragments are homologous to 60 bp just up- and downstream of the *ACS2* ORF, respectively, thus enabling repair of the Cas9-induced double strand break by HR (left panel). Deletion of *ACS1* using a 120 bp dsDNA repair fragment is shown in the right panel. (**b**) Multiplex colony PCR was performed on 10 transformants to check their genotypes. Results are shown for two representative colonies, confirming the intended genotype. The PCR on the wild-type strain (CEN.PK113-7D) shows the predicted bands for the presence of the wild-type *ACS1* and *ACS2* alleles. Two DNA ladders were used; L1 refers to the GeneRuler DNA ladder (Thermo Scientific) and L2 to the GeneRuler 50 bp DNA ladder (Thermo Scientific). (The bands indicated with an asterisk reflect aspecific PCR products).

#### Single gRNA method (cloning-free)

The yeast HR machinery was used to assemble plasmids with specific gRNA sequences out of two different fragments: a plasmid backbone and a gRNA target sequence. Depending on the preferred selectable marker, the linearized plasmid backbone was obtained via PCR using the appropriate single gRNA plasmid (pMEL10–pMEL17) as a template, with primers 6005 & 6006. To obtain the double-stranded gRNA cassettes (the target sequences are listed in Table 3), two complementary single-stranded oligos (Table S1, Supplementary data) were mixed in a 1:1 molar ratio, heated to 95°C and then cooled down to room temperature. The resulting gRNA fragments contained the 20 bp gRNA recognition sequences, flanked by 50 bp overlaps with the linearized plasmid backbone. The 120 bp repair fragments were obtained by following the same procedure and were identical to the up- and downstream regions of the DSB break, allowing for effective repair by the HR-machinery. For each transformation, a linearized plasmid backbone, a double-stranded cassette containing the gRNA sequence of choice and the double-stranded DNA cassette for repair of the DSB were pooled and co-transformed to the appropriate strain.

**Table 3.** Target sequences used in this study.

Locus	Sequence (including PAM)	Restriction site	AT score	RNA score
*MCH1*	TATTGGCAATAAACATCTCGAGG		0.65	0.50
*MCH2*	ATCTCGATCGAGGTGCCTGATGG	*Pvu*I	0.45	0.30
*MCH5*	ACTCTTCCGTTTTAGATATCTGG	*Eco*RV	0.65	0.50
*AQY1*	ACCATCGCTTTAAAATCTCTAGG	*Dra*I	0.65	0.50
*ITR1*	ATACATCAACGAATTCCAACCGG	*Eco*RI	0.65	0.60
*PDR12*	GCATTTTCGGTACCTAACTCCGG	*Kpn*I	0.55	0.65
*NAT1*	AAAGGAATTGGATCCTGCGTAGG	*Bam*HI	0.55	0.60
*GET4*	GGGCTCGCTAGGATCCAATTCGG	*Bam*HI	0.45	0.50
*ACS1*	TTCTTCACAGCTGGAGACATTGG	*Pvu*II	0.55	0.45
*ACS2*	TCCTTGCCGTTAAATCACCATGG		0.55	0.75

#### Double gRNA method

Plasmids with two gRNAs were assembled *in vitro*, using Gibson assembly of a 2 μm fragment containing the two gRNA sequences and a double gRNA (pROS10–pROS17) plasmid backbone. The 2 μm fragment was obtained via PCR, using pROS10 as a template with two primers containing the 20 bp gRNA recognition sequences and a 50 bp sequence, homologous to the linearized plasmid backbone (Table S1, Supplementary data). The linearized plasmid backbone was obtained via PCR using one of the double gRNA plasmids (pROS10–pROS17, depending on the preferred selective marker) as a template with a single primer (6005), binding at each of the two *SNR52* promoters (Supplementary data). The two fragments were combined using Gibson assembly, followed by transformation to *E. coli* for storage and plasmid propagation. Since both of the gRNA containing primers could bind on either side of the 2 μm fragment, it was important to check that the final plasmid contained one copy of each gRNA (theoretically this would be the case in 50% of the *E. coli* transformants). To simplify this confirmation step, the gRNA target sequences were selected for the presence of a restriction site. Alternatively, diagnostic primers specific for the introduced 20 bp recognition sequences were used for the identification of correctly assembled gRNA plasmids using PCR (see Protocol in Supplementary data).

To construct the plasmid pUDR002 (targeting *MCH1&MCH2*, *TRP1* marker), the 2 μm fragment was amplified using DreamTaq (Fisher Scientific) from pROS10 using primers 6835 & 6837 (Supplementary data). The backbone of pROS17 was amplified using the Phusion polymerase (Fisher Scientific) with a single primer 6005 (Supplementary data). The two fragments were assembled using Gibson assembly and confirmed via restriction analysis. Similarly, the following plasmids were constructed: pUDR004 (targeting *MCH5&AQY1, HIS3* marker), pUDR005 (targeting *ITR1&PDR12, KlURA3* marker), pUDR020 (targeting *NAT1*&*GET4, URA3* marker) and pUDR022 (targeting *ACS1*&*ACS2*, *kanMX* marker). Transformations using the double gRNA method required co-transformation of 2 μg of (each) pUDR plasmid together with 1 μg of (each) corresponding double-stranded DNA cassette for DSB repair.

### Molecular biology techniques

PCR amplification with Phusion® Hot Start II High Fidelity Polymerase (Thermo Fisher Scientific) was performed according to the manufacturer's instructions using PAGE-purified oligonucleotide primers (Sigma-Aldrich, St. Louis, MO, USA). Diagnostic PCR was done via colony PCR on randomly picked yeast colonies, using DreamTaq (Thermo Fisher Scientific) and desalted primers (Sigma-Aldrich). The primers used to confirm successful deletions by one of the two described methods can be found in Table S1 (Supplementary data). DNA fragments obtained by PCR were separated by gel electrophoresis on 1% (w/v) agarose gels (Thermo Fisher Scientific) in TAE buffer (Thermo Fisher Scientific) at 100 V for 30 minutes. Fragments were excised from gel and purified by gel purification (Zymoclean™, D2004, Zymo Research, Irvine, CA, USA). Plasmids were isolated from *E. coli* with Sigma GenElute Plasmid kit (Sigma-Aldrich) according to the supplier's manual. Yeast plasmids were isolated with Zymoprep Yeast Plamid Miniprep II Kit (Zymo Research). *Escherichia coli* DH5α (18258–012, Life Technologies) was used for chemical transformation (T3001, Zymo Research) or for electroporation. Chemical transformation was done according to the supplier's instructions. Electrocompetent DH5α cells were prepared according to Bio-Rad's protocol, with the exception that the cells were grown in LB medium without NaCl. Electroporation was done in a 2 mm cuvette (165–2086, Bio-Rad, Hercules, CA, USA) using a Gene Pulser Xcell Electroporation System (Bio-Rad), following the manufacturer's protocol.

### Yeastriction webtool

The tool is written in Javascript and based on the MEAN.io stack (MongoDB, Express, AngularJS and Node.js). The source code is available at https://github.com/hillstub/Yeastriction. Genome and ORF sequences were downloaded from SGD (http://www.yeastgenome.org) in GFF and FASTA file format, respectively. ORFs, including their 1 kb up- and downstream sequences, were extracted and imported into Yeastriction, with the aid of an in-house script.

Yeastriction extracts all possible Cas9 target sequences (20 bp followed by NGG) from a specified ORF and from its complementary strand. Subsequently, sequences containing six or more Ts are discarded as this can terminate transcription (Braglia, Percudani and Dieci 2005; Wang and Wang 2008). Target sequences are then tested for off-targets (an off-target is defined as a sequence with either the NGG or NAG PAM sequence and 17 or more nucleotides identical to the original 20 bp target sequence; Hsu *et al.* 2013) by matching the sequences against the reference genome using Bowtie (version 1) (Langmead *et al.* 2009). If any off-target is found, the original target sequence is discarded. In a next step, the AT content is calculated for the target sequence. Using the RNAfold library (essentially with the parameters –MEA –noLP –temp = 30.) (Lorenz *et al.* 2011), the maximum expected accuracy structure of each RNA molecule is calculated. The target sequence is also searched for the presence of restriction sites based on a default list or a user-defined list. The targets can be ranked based on the presence of restriction sites (1 for containing and 0 for lacking a restriction site), AT content (1 having the highest AT content and 0 for the lowest AT content) and secondary structure (1 having the lowest amount of pairing nucleotides and 0 for the highest number of nucleotides involved in secondary structures, indicated by brackets). The range for every parameter is determined per locus and used to normalize the values. Subsequently, the target sequences are ranked by summation of the score for each parameter. These ranking scores should only be used to order the targets from a single locus and not to compare targets for different loci. The application can be accessed at the following URL: http://yeastriction.tnw.tudelft.nl/.

## RESULTS AND DISCUSSION

### Yeastriction: a CRISPR design tool

To streamline design and construction of CRISPR/Cas9 gRNA plasmids for introduction of (multiple) genetic modifications, the Yeastriction webtool (http://yeastriction.tnw.tudelft.nl) was developed. This webtool was designed to be compatible with the single and double gRNA plasmid series (pMEL10–pMEL17 and pROS10–pROS17, respectively), as described below. Because the CRISPR/Cas9 system can be highly sequence specific, it is crucially important to select target sequences based on correct reference genome sequence information. A difference of a single nucleotide in the gRNA, as compared to the genomic target sequence, can already completely abolish Cas9 nuclease activity (Hsu *et al.* 2013). To make Yeastriction useful for the entire yeast community, a set of 33 *S. cerevisiae* genomes (www.yeastgenome.org) was implemented. In the first step, the user can select the desired reference genome and enter (multiple) systematic names (e.g. YDL054C, YKL221W) or gene names (e.g. *MCH1*, *MCH2*). For each entered gene, the tool matches every potential target sequence to the selected reference genome. If there are potential off-targets (other sequences present in the same genome with either NAG or NGG as PAM sequence and with a 0–3 nucleotide difference in the 20 bp target sequence, Hsu *et al.* 2013), the target sequence is discarded. Sequences that contain six or more consecutive Ts are also discarded as they may cause transcript termination (Braglia, Percudani and Dieci 2005; Wang and Wang 2008). A recent report indicates that the AT content of Cas9 target sequences should preferably be above 65% (Lin *et al.* 2014). Furthermore, there are indications that target sequences without obvious nucleotide interactions in secondary structures are more efficient (Zhang 2014). The presence of unique restriction sites within the target sequence simplifies verification of correct plasmid assembly. Yeastriction therefore ranks potential Cas9 target sequences according to AT content, secondary structures and the presence of restriction sites. To increase flexibility, the user can also choose to leave out one of the parameters in the final ranking. For the top-ranked target sequence, the tool automatically designs the oligonucleotide primers required for plasmid construction and the oligonucleotides (which can be ordered as primers) needed to form the repair fragment when a gene deletion is desired. To increase flexibility, the user can also choose another target site than the top-ranked sequence (Supplementary data). In comparison with existing tools such as ChopChop (Montague *et al.* 2014), Yeastriction combines several features to improve gRNA sequence selection for *S. cerevisiae*: (i) yeast strain specificity, (ii) elimination of spacer sequences with potential off-targets, (iii) ranking of the remaining gRNA sequences based on RNA structure and AT content and (iv) direct generation of primer sequences, compatible with the transformation methods described below.

### Construction of a set of plasmids for transformation with one or two gRNAs

DiCarlo *et al.* (2013b) described construction of plasmid p426-SNR52p-gRNA.CAN1.Y-SUP4t (Addgene www.addgene.org/43803/) for expression of a single gRNA in yeast, using the *SNR52* promoter and *SUP4* terminator. We hypothesized that the gRNA could be easily changed by *in vivo* HR via co-transformation of the linearized p426-SNR52p-gRNA.CAN1.Y-SUP4t backbone (from which the 20 bp recognition sequence ‘*CAN1*.Y’ was omitted) together with a new 20 bp gRNA fragment, flanked by 50 bp overlaps with the plasmid backbone. In order to further increase the flexibility of this system and to allow its use with genetic markers other than *URA3*, a standardized set of plasmids was constructed (Fig. 1B). To this end, a linearized plasmid backbone of the p426-SNR52p-gRNA.CAN1.Y-SUP4t plasmid that excluded the *URA3* marker gene was obtained by PCR. Subsequently, eight different genetic markers (*KlURA3, amdSYM, hphNT1, kanMX, KlLEU2, natNT2, HIS3* and *TRP1*) were PCR amplified and (re-)introduced in this backbone using Gibson assembly. The resulting plasmids were named pMEL10 to pMEL17 (Fig. 1B, Table 2)

The use of *in vivo* recombination enabled the introduction of one gRNA sequence per single gRNA plasmid backbone (pMEL10–pMEL17), without the need of prior cloning. When multiple genetic modifications are required, marker availability might become a limitation. In this case, plasmids containing multiple gRNA sequences would be preferred as this allows introduction of more genetic modifications before plasmid removal is required. For this purpose, a second set of plasmids was constructed that contained two gRNA sequences, between separate promoters and terminators.

First, pROS10 was constructed (Fig. 1C), containing the *URA3* marker, ampR for selection in *E. coli* and a 2 μm fragment flanked by two p*SNR52*-gRNA-t*SUP4* cassettes in opposite directions, with one of these cassettes containing the target sequence *CAN1*.Y and the other *ADE2*.Y (DiCarlo *et al.* 2013b). This design enables construction of plasmids that target other loci, by first PCR amplifying the 2 μm fragment, using primers that incorporate the new target sites, followed by assembly into a linearized backbone that contains the desired marker gene via an *in vitro* method such as Gibson assembly. To generate plasmids with marker genes other than *URA3*, the backbone of pROS10 was first digested with restriction enzymes to remove the *URA3* marker. Subsequently, other markers were PCR amplified and inserted using Gibson assembly. This yielded plasmids pROS11 to pROS17 (Fig. 1C, Table 2), which contained the marker genes *amdSYM, hphNT1, kanMX, KlLEU2, natNT2, HIS3 and TRP1*, respectively. Both plasmid series (pMEL10–pMEL17 and pROS10–pROS17) have been made available through EUROSCARF.

### Construction of various cas9-expressing CEN.PK strains

In the *cas9*-bearing strains described by DiCarlo *et al.* (2013b), a centromeric plasmid was used to express *cas9*, either from a variant of the inducible *GAL1* promoter or from the constitutive *TEF1* promoter. When multiple rounds of transformation and gRNA plasmid removal are desired, a stable integrated copy of *cas9* is preferred, since this allows growth of strains on complex medium, which enables faster growth and efficient plasmid recycling. To this end, a *cas9* gene under the control of the *TEF1* promoter was integrated into the *CAN1* locus together with the *natNT2* marker. In order to increase the flexibility of the CRISPR/Cas9 system, *cas9* was integrated in the haploid strains CEN.PK113–7D and CEN.PK113–5D (Ura*^−^*) and the diploid strains CEN.PK122 and CEN.PK115 (Ura^−^). Additionally, *cas9* was integrated in the quadruple auxotrophic strain CEN.PK2–1C (Ura^−^, Leu^−^, Trp^−^, His^−^), resulting in strain IMX672, which was used to test the efficiencies of the gRNA plasmids developed in this study. All these strains are deposited at EUROSCARF. Constitutive expression of *cas9* in CEN.PK113–7D (IMX585) did not affect the maximum specific growth rate. IMX585 grew at 0.37 ± 0.003 h^−1^ (value and mean deviation are based on two independent experiments) on glucose SM in shake-flask cultures while the growth rate of the reference CEN.PK113–7D was 0. 39 ± 0.01 h^−1^.

### Seamless and markerless gene deletion using *in vivo* assembled plasmids containing single gRNAs

The first plasmid series, pMEL10–pMEL17, was designed to perform gene deletions from plasmids assembled *in vivo*, harnessing the high frequency and fidelity of HR in *S. cerevisiae* (Orr-Weaver and Szostak 1983; Orr-Weaver, Szostak and Rothstein 1983; Kunes, Botstein and Fox 1985; Kuijpers *et al.* 2013b) and obviating the need for prior cloning of the gRNA sequence. To test whether *in vivo* assembly of a plasmid carrying the gRNA sequence could be combined with CRISPR-mediated genetic modification, a plasmid backbone and insert containing the gRNA of choice were co-transformed with a 120 bp ‘repair fragment’ for markerless and scarless gene deletion. The gRNA sequence targeting the gene *MCH1* (Table 3) was selected based on a high score (AT content 0.65, RNA score 0.50) in Yeastriction and contained 50 bp overlaps to each side of the linearized plasmid backbone with the *KlURA3* marker, facilitating HR. Transformation of IMX672 (*ura3–52 trp1–289 leu2–3,112 his3Δ*, *can1Δ::cas9*) resulted in >5000 colonies per plate and colony PCR was performed to confirm successful gene deletion. Out of 24 randomly tested colonies, 12 (50%) showed the intended single gene deletion of *MCH1* (Fig. 2). The colonies that did not show the intended deletion could be caused by misassembly of the plasmid, as omitting either the insert or the repair fragment from the transformation mixture yielded ∼2000 colonies. To test whether these results could be reproduced when different loci were targeted, the same strategy was employed to target *MCH2* and *MCH5*. A gRNA recognition sequence was selected for each gene using Yeastriction, based on a low score for *MCH2* (AT content 0.45, RNA score 0.30) and a high score for *MCH5* (AT content 0.65, RNA score 0.50) targeting sequence, respectively. Transformation with gRNA inserts targeting either of these loci and the corresponding repair fragments resulted in similar colony counts as observed for *MCH1*. Furthermore, colony PCR determined successful deletions for both loci at varying efficiencies, 25% and 75% for *MCH2* and *MCH5*, respectively (Fig. 2). This observed difference in deletion efficiency was in line with the quality scores predicted by Yeastriction for *MCH2-* and *MCH5*-selected gRNAs. This cloning-free approach might be extended to enable co-transformation of multiple gRNA inserts and their corresponding repair fragments. By assembly of multiple plasmids bearing different gRNA sequences in a single cell, this might facilitate the simultaneous introduction of multiple gene deletions. To test this possibility, the same plasmid backbone, containing the *KlURA3* marker, was co-transformed with three different gRNA fragments (*MCH1*, *MCH2* & *MCH5*) and their corresponding repair fragments. Over 5000 colonies were obtained after transformation and 24 randomly picked colonies were tested via colony PCR. Transformation with three inserts led to the identification of four mutants containing a single and one mutant containing a double deletion (24 colonies tested, Fig. 2A) but none with three deletions. Only one of the six identified deletions was found in the *MCH1* locus (targeted by a gRNA with a low Yeastriction score, Table 3), while three and two deletions corresponded to ORFs of *MCH1* and *MCH5* respectively. These results indicated that mutants containing multiple gene deletions could be obtained via combined *in vivo* recombination and gene disruption, although at low (∼4%) efficiencies. This low efficiency of multiple gene disruption might have several reasons: (i) it can be the result of misassembly of the plasmid, as transformation of the backbone without insert or repair oligo already resulted in ∼2000 colonies when making single deletions; (ii) a cell does not need to assemble all three plasmids, as one plasmid is enough to restore prototrophy; and (iii) errors present in the gRNA inserts affecting its targeting efficiency. Indeed, sequencing of *in vivo* assembled plasmids of three false positive colonies showed that the gRNA sequence contained nucleotidic insertions and deletions, impairing *cas9* restriction in the target ORF. These mutations of the gRNA sequence likely derived from imperfect annealing of the two complementary oligonucleotides used to form the *in vivo* assembled gRNA fragment.

All collectively these results showed that single gene deletions could be easily performed by this method; however, when more than one locus has to be altered a more effective strategy is needed as is detailed in the next section.

### Multiplexing seamless gene deletions using *in vitro* assembled plasmids containing two gRNAs

While in *vivo assembly* of the gRNA plasmid and CRISPR-assisted genetic modification can be combined in a single transformation, the relatively high incidence of false positive colonies limits the use of this system for simultaneous introduction of multiple chromosomal modifications. Since some of the false positives could be the result of misassembly of the gRNA containing plasmid, it was expected that pre-assembly of the plasmid using *in vitro* Gibson assembly would result in a lower number of false positives. Therefore, a double gRNA plasmid was constructed, expressing guide RNAs designed to target *ITR1* and *PDR12*. First, the pROS10 backbone and the 2 μm fragment flanked by the gRNAs containing the target sequences for *ITR1* and *PDR12* were amplified using PCR (Fig. 1). The resulting DNA fragments were assembled and the plasmid (pUDR005) was verified by digestion with restriction enzymes specifically targeting the *ITR1* and *PDR12* target sequences (Table 3). Co-transformation of the resulting plasmid with the corresponding repair fragments into the *cas9-*expressing strain IMX672 yielded 900 colonies per plate. In contrast, a single transformation in which repair fragments were omitted yielded only two colonies. 24 colonies were randomly picked and PCR verification determined that all of these contained both gene disruptions (IMX715, Fig. 3). The transformation of an already assembled plasmid might enable immediate transcription of the gRNA, while the other method required assembly of a correct plasmid prior to transcription of the gRNA. This might explain the significantly higher observed efficiency of this approach compared to the method using *in vivo* assembled plasmids. Most likely, pre-assembly and confirmation of the plasmid containing two gRNAs greatly reduced the number of false positives.

Encouraged by these extremely high efficiencies, transformations were performed using multiple plasmids that carried different genetic markers. To this end, two new plasmids were constructed, each targeting two different genetic loci [*MCH1* & *MCH2* (pUDR002) and *MCH5* & *AQY1* (pUDR004)]. Two and three plasmids were co-transformed, containing either the *KlURA3* and *TRP1* or the *KlURA3*, *TRP1* and *HIS3* selectable markers. In contrast to the transformations with a single plasmid, which yielded over 900 colonies per transformation plate, only 74 colonies and 14 colonies were obtained when transforming two and three plasmids, respectively. These lower numbers of colonies might reflect the decreasing probability that a single cell successfully takes up multiple plasmids and, subsequently, performs the corresponding gene deletions. The characterization of the colonies from the transformation with two plasmids revealed that out the 20 tested clones, 14 (70%) harboured all 4 deletions (IMX716). Similarly, out of 20 randomly tested colonies obtained from the transformations with 3 plasmids, 13 (65%) clones contained all 6 deletions (IMX717, Fig. 3).

These results unequivocally demonstrate the efficiency of CRISPR/Cas9-mediated genetic modification of yeast in simultaneously generating multiple deletions in a single transformation step. Recently, CRISPR/Cas9 was successfully applied to simultaneously disrupt all homozygous alleles in the polyploid ATCC4124 strain. In four transformation iterations, a quadruple *ura3 trp1 leu2 his3* auxotrophic strain was constructed (Zhang *et al.* 2014). A design similar to the native polycistronic CRISPR array consisting of a tracrRNA and crRNA instead of the chimeric gRNA was used to achieve three concurrent deletions using a single crRNA array (Bao *et al.* 2014). To the best of our knowledge, the present study is the first to demonstrate generation of a sextuple deletion strain of *S. cerevisiae* in a single transformation event.

### Multiplexing deletion, multigene integration and introduction of single nucleotide mutations

Hitherto, reported applications of CRISPR/Cas9 in *S. cerevisiae* focused on gene inactivation. I-SceI-mediated introduction of double-stranded breaks has previously been shown to facilitate simultaneous integration of several gene expression cassettes at chromosomal loci (Kuijpers *et al.* 2013a). To explore the potential of CRISPR to combine gene deletion with the simultaneous *in vivo* assembly and chromosomal integration of multiple DNA fragments, we attempted to construct a *S. cerevisiae* strain with a double *ACS1 ACS2* deletion that also overexpresses the *E. faecalis* pyruvate dehydrogenase (PDH) complex (Kozak *et al.* 2014b) in a single transformation. To this end, IMX585 was transformed with a plasmid expressing the gRNAs targeting the *ACS* genes (pUDR022). A 120 bp repair fragment was co-transformed for the deletion of *ACS1*, and the ORF of *ACS2* was replaced via integration of six gene cassettes expressing the genes of the E1α, E1β, E2 and E3 subunits of *E. faecalis* PDH encoded by *pdhA*, *pdhB*, *aceF*, and *lpd*, as well as the *lplA* and *lplA2* genes required for PDH lipoylation (Kozak *et al.* 2014b) (Fig. 4A). Cytosolic acetyl-CoA is essential and a double *acs1*Δ *acs2*Δ mutant is not viable (van den Berg *et al.* 1996) unless acetyl-CoA is provided by an alternative route (Kozak *et al.* 2014a,b). The transformation targeting the two *ACS* genes yielded 11 colonies, with 10 out of 10 picked colonies showing the desired genotype (Fig. 4B). From one colony, the gRNA plasmid was removed by growing on non-selective YP medium, yielding strain IMX719. This strain showed only growth on SM with 2% glucose and lipoate and failed to grow on SM with 2% glucose and on YP with 2% ethanol. This phenotype is consistent with the phenotype reported by Kozak *et al.* (2014b) for an *acs1 acs2* strain of *S. cerevisiae* that expresses the *E. faecalis* PDH subunits and lipoylation genes, thereby further confirming the genotype of the strain IMX719.

**Figure 5.**
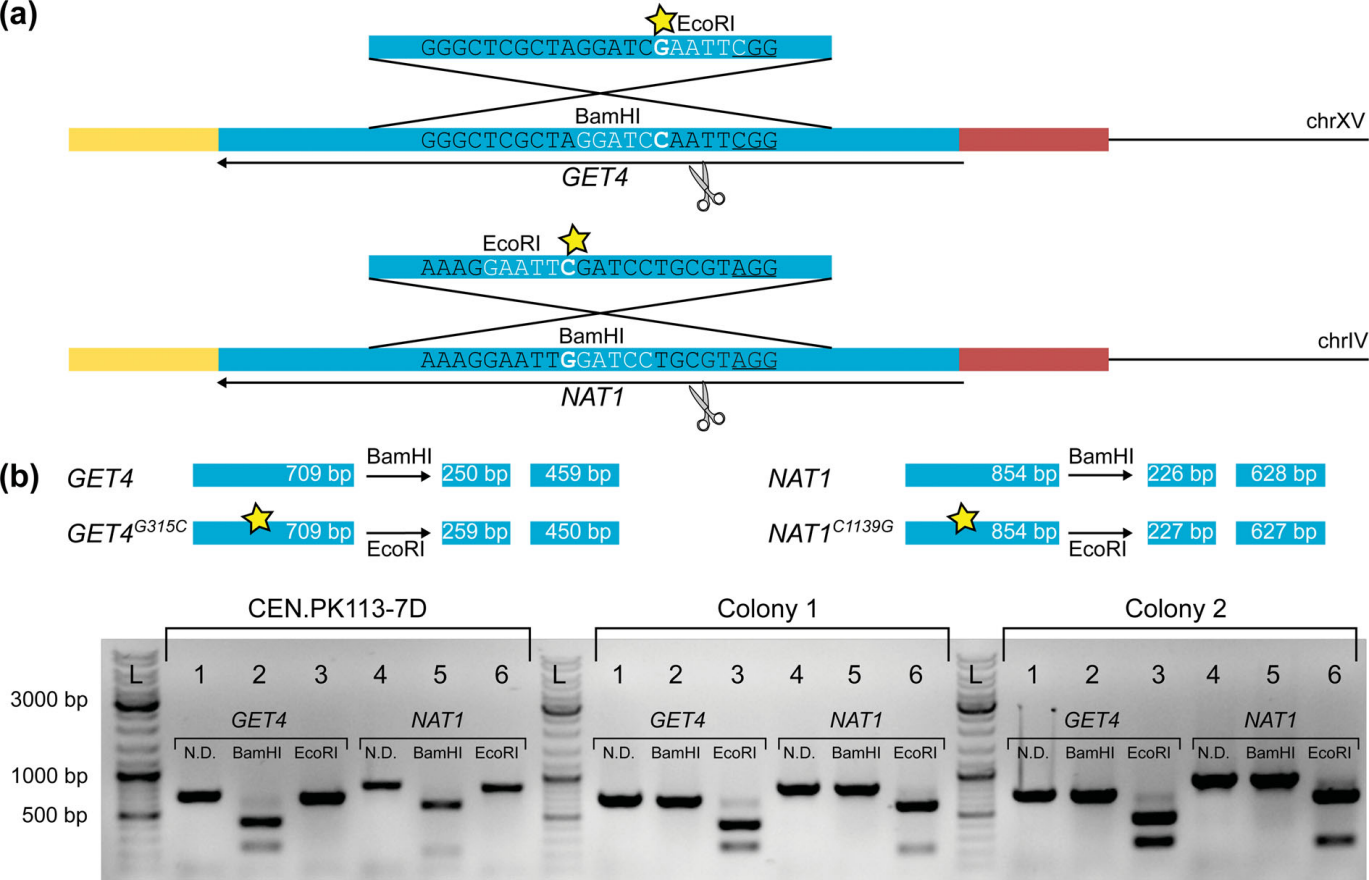
Simultaneous introduction of different single-nucleotide mutations in *S. cerevisiae*. **(a)** Transformation of IMX581 (*ura3–52, can1Δ::CAS9*) was performed with pUDR020, resulting in the introduction of two mutations in *NAT1* and *GET4*. Underlined: the PAM sequences associated with the gRNA targets. In white: restriction sites present in the original gRNA targeting sequence (*Bam*HI) and in the repair fragment used to correct the double-strand break (*Eco*RI). (**b**) Introduction of the double-strand break and subsequent repair using the mutagenic repair fragment resulted in a change of restriction site from *Bam*HI to *Eco*RI. In this colony PCR example, primers 7030 & 7031 and 7036 & 7037 were used to amplify a part of the *GET4* and *NAT1* locus (lane 1 and 4, respectively) from CEN.PK113–7D and two colonies of IMX581, transformed with pUDR020 and mutagenic repair fragments. In lanes labelled 2 and 5: digestion of the PCR fragments with *Bam*HI, which only results in digestion fragments of sizes 250 bp & 459 bp and 226 bp & 628 bp when the original restriction sites in *GET4* and *NAT1* are still present (CEN.PK113–7D). In lanes labelled 3 and 6: digestion of the PCR fragments with *Eco*RI, which only results in digestion fragments of sizes 259 bp & 450 bp and 227 bp & 627 bp when this new restriction site has been introduced via the mutagenic repair fragment (colony 1 and 2).

To explore the potential of CRISPR/Cas9 to introduce specific single-nucleotide mutations in genomic DNA, a plasmid was constructed with gRNAs targeting the *GET4* and *NAT1* loci (pUDR020). Both of the target sequences contained a *Bam*HI (G|GATCC) restriction site. The corresponding repair oligonucleotides were designed to introduce a single-nucleotide change in the genomic target sequence, which at both sites resulted in the introduction of a restriction site for *Eco*RI (G|AATTC) and simultaneously disrupted the *Bam*HI restriction site (Fig. 5A). Transformation of the *cas9*-bearing strain IMX672 with pUDR020 (*NAT1* and *GET4* gRNA, *KlURA3*) and the corresponding repair fragments resulted in ∼1500 colonies, while omitting both repair fragments did not result in any colonies. Eight colonies were randomly picked and a part of the ORF containing the target sequence was PCR amplified for both *GET4* and *NAT1*. Digestion of the amplified PCR product with *Bam*HI and *Eco*RI, followed by gel electrophoresis, showed that four of the eight colonies contained both mutations after transformation (Fig. 5B). To confirm whether these four colonies indeed contained the desired mutations, a fragment of 120 bp around the target site was sequenced. All colonies contained the desired mutations, although two of the four colonies also showed additional, undesired, mutations at the *GET4* locus (supplementary data S1, colony 2 and 4). Although these results show that one mutation can already abolish restriction, Cas9-induced DSBs might still occur, as long as the gRNA cassette is expressed. We therefore advise to (re)sequence mutated sites after gRNA plasmid removal and/or use the two-step strategy discussed below.

The ease with which specific single-nucleotide mutations can be simultaneously introduced makes the CRISPR/Cas9 system a highly valuable tool for analysing the biological significance of mutations identified by whole-genome resequencing of strains obtained by mutagenesis (e.g. UV, X-rays) (Jung *et al.* 2011) or evolutionary engineering (Hong *et al.* 2011; González-Ramos *et al.* 2013; Oud *et al.* 2013; Caspeta *et al.* 2014). Reverse engineering of such mutations essentially encompasses restoration of the reference nucleotide in the mutant strain and/or introduction of the mutated nucleotide position in a naïve (non-mutated or non-evolved) genetic background. Even laboratory evolution experiments performed in the absence of mutagenesis typically yield multiple mutations, not all of which contribute to the phenotype of interest (see e.g. de Kok *et al.* 2012; González-Ramos *et al.* 2013). Therefore, availability of methods that enabled reintroduction of multiple point mutations at various genomic loci is invaluable for rapid identification of relevant mutations. Similar to the YOGE (Yeast Oligo-Mediated Genome Engineering) method (DiCarlo *et al.* 2013a), the CRISPR approach enables multiplexing and offers flexibility. In contrast to the YOGE method, which requires a specific background (*mlh1*Δ *msh2*Δ *RAD51^K342E^*↑ *RAD54*↑) (DiCarlo *et al.* 2013a), the CRISPR approach should be applicable to any *S. cerevisiae* strain background that expresses a functional *cas9* gene. The two examples used in this study (*GET4* and *NAT1*) were designed for easy verification. If the desired point mutation is not present in a suitable target sequence, it may be possible to introduce (multiple) single nucleotide variation(s) (SNV) in two rounds of CRISPR/Cas9-mediated genome modification. In the first round, a larger part surrounding one or several SNVs could be deleted while repairing the DSBs with repair fragments that contain a generic synthetic target sequence. In a second round, these generic target sequences can then be cut by Cas9, combined with the repair of the DSBs with 120 bp sequences that contain the desired SNVs.

### Outlook

The use of HR for the assembly of multigene constructs (Gibson *et al.* 2008; Shao, Zhao and Zhao 2009) had a tremendous impact on genetic engineering strategies (Annaluru *et al.* 2014; Casini *et al.* 2014). Even before the advent of CRISPR/Cas9, these developments have immensely decreased strain construction time in our group and enabled us to express complicated multistep pathways and multicomponent enzymes (Koopman *et al.* 2012; Guadalupe-Medina *et al.* 2013; Kuijpers *et al.* 2013a; Beekwilder *et al.* 2014; Kozak *et al.* 2014b). Until recently, the deletion of multiple genes and insertions at multiple loci remained a cumbersome and time-intensive process. Here, building on the pioneering conceptual proof of CRISPR functionality in lower eukaryotes (DiCarlo *et al.* 2013b), we show that CRISPR/Cas9 will further improve and accelerate yeast strain construction, not only by allowing multiplexed gene deletions (DiCarlo *et al.* 2013b; Zhang *et al.* 2014) but also by allowing simultaneous introduction of gene deletions, chromosomal integration of multigene constructs and the introduction of specific mutations. Although it is difficult to quantify the impact on the overall time requirements for complex pathway engineering, the examples presented here suggest that a 3- to 4-fold acceleration is unlikely to be exaggerated.

This paper focuses on *S. cerevisiae*, a microbial species that is already known for its easy genetic accessibility. Therefore, it is logical to speculate that this methodology should have an even higher impact on species of yeasts and filamentous fungi that are notoriously more difficult to alter genetically. Indeed, a very recent example described the benefit of the introduction of CRISPR/Cas9 in *Schizosaccharomyces pombe* (Jacobs *et al.* 2014). While the methodology reported in this study streamlines the use of CRISPR in *S. cerevisiae*, the method can be further improved. The RNA polymerase III-dependent promoter *SNR52* is not a broadly recognized promoter (Ryan *et al.* 2014). Recently, it has been shown that guideRNAs, flanked by the hammerhead and hepatitis delta virus ribozymes, can be expressed using polymerase II promoters (Gao and Zhao 2014). A promising promoter would then be the *TEF1* promoter from *Blastobotrys adeninivorans* (Terentiev *et al.* 2004) as this is a strong, constitutive promoter, recognized in different species, like *S. cerevisiae*, *Hansenula polymorpha* and *Pichia pastoris*.

We hope that by providing the easy-to-use Yeastriction design tool, two versatile plasmid series for gRNA expression, a set of *S. cerevisiae* CEN.PK strains harbouring the *cas9* expression cassette and standardized protocols (Supplementary data), this paper will help colleagues to facilitate and accelerate yeast strain engineering.

## SUPPLEMENTARY DATA

Supplementary data is available at FEMSYR online.

Supplementary data is available at FEMSYR online
