# Supplementary material for: CRISPR/Cas9: a molecular Swiss army knife for simultaneous introduction of multiple genetic modifications in Saccharomyces cerevisiae
Source: FEMS Yeast Res. 2015 Mar 17;15(2):fov004. doi: 10.1093/femsyr/fov004 (PMC4399441; doi:10.1093/femsyr/fov004)
Supplement: Supplementary data is available at FEMSYR online [file femsyr_fov004_index.html]

SUPPLEMENTARY DATA | FEMS Yeast Research

## SUPPLEMENTARY DATA

**Files in this Data Supplement:**

- SUPPLEMENTARY DATA
