# Supplementary material for: CRISPR/Cas9: a molecular Swiss army knife for simultaneous introduction of multiple genetic modifications in Saccharomyces cerevisiae
Source: FEMS Yeast Res. 2015 Mar 17;15(2):fov004. doi: 10.1093/femsyr/fov004 (PMC4399441; doi:10.1093/femsyr/fov004)
Supplement: Supplementary data is available at FEMSYR online [file Supplemental_information.pdf]

Supplemental information

Table of Contents

Tutorial Yeastriction 2
Protocol for gene editing using in vivo assembled plasmids containing single gRNAs 6
Protocol for multiplexed gene editing using in vitro assembled plasmids containing two gRNAs 10
Supplementary Table S1 17
Supplementary Data S1 21

Supplemental information 1
Tutorial Yeastriction

If desired, first sign up to the webservice by going to http://yeastriction.tnw.tudelft.nl and clicking Register in the top right corner
(the advantage of registering is that the webtool can save your defaults like your strain of choice, restriction enzymes, etc.).
After that, login using the Login link, also in the top right corner. The following screen will be shown.

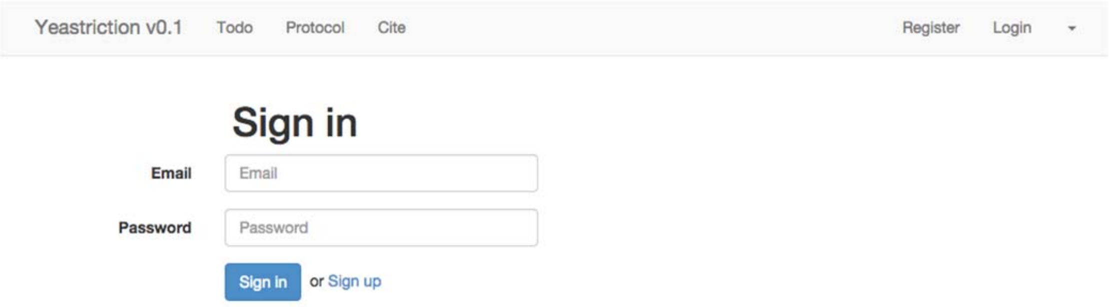

After logging in, click Yeastriction to start using the tool. In the screen (see below) select your yeast strain in the dropdown.

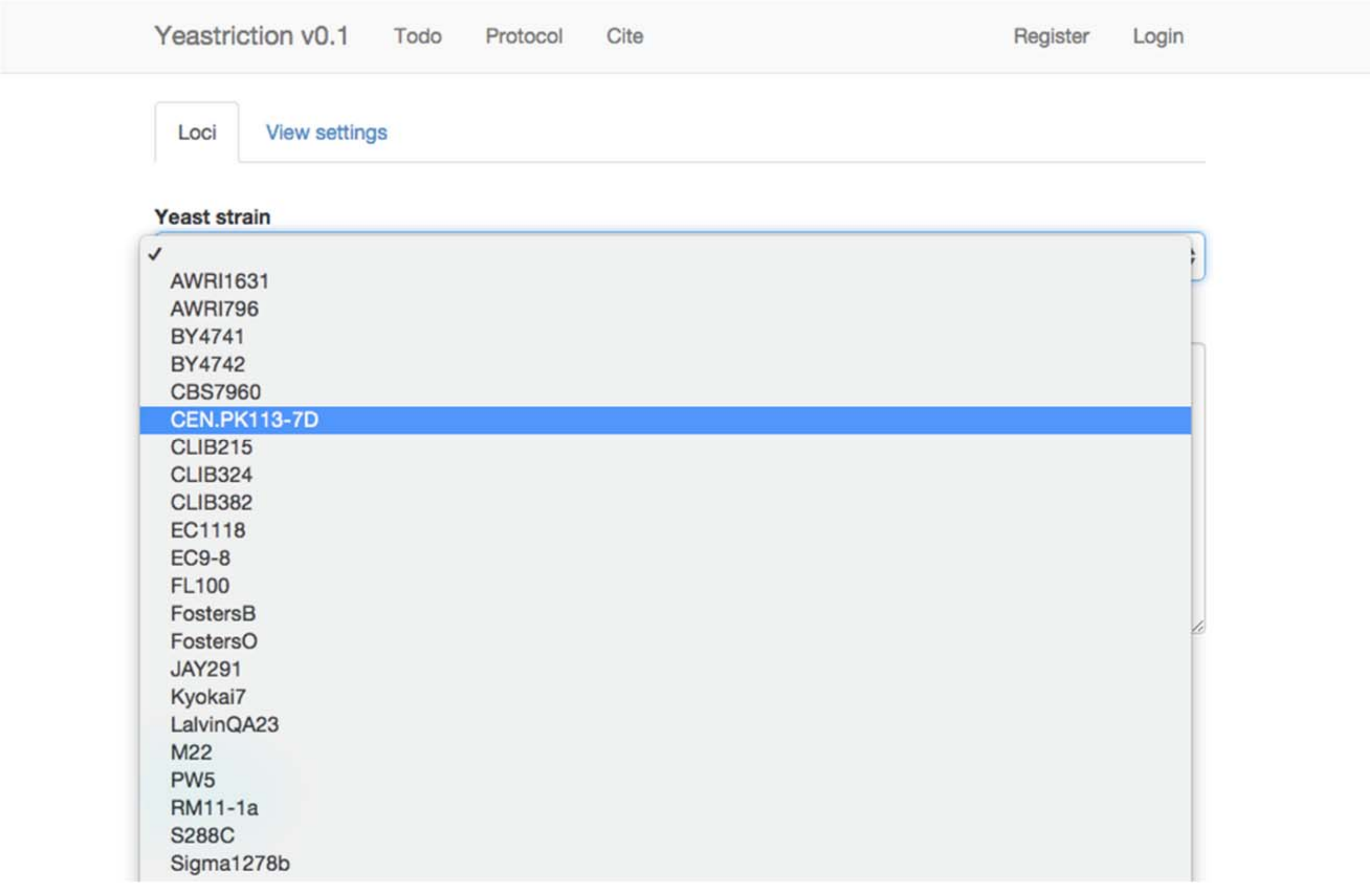

Next, write one or multiple gene names (e.g. *ACS1 ACS2*) and/or systematic names (like YAL054C YLR153C).
(The delimiter between the names doesn't matter; it can be spaces, commas, newlines, etc.)

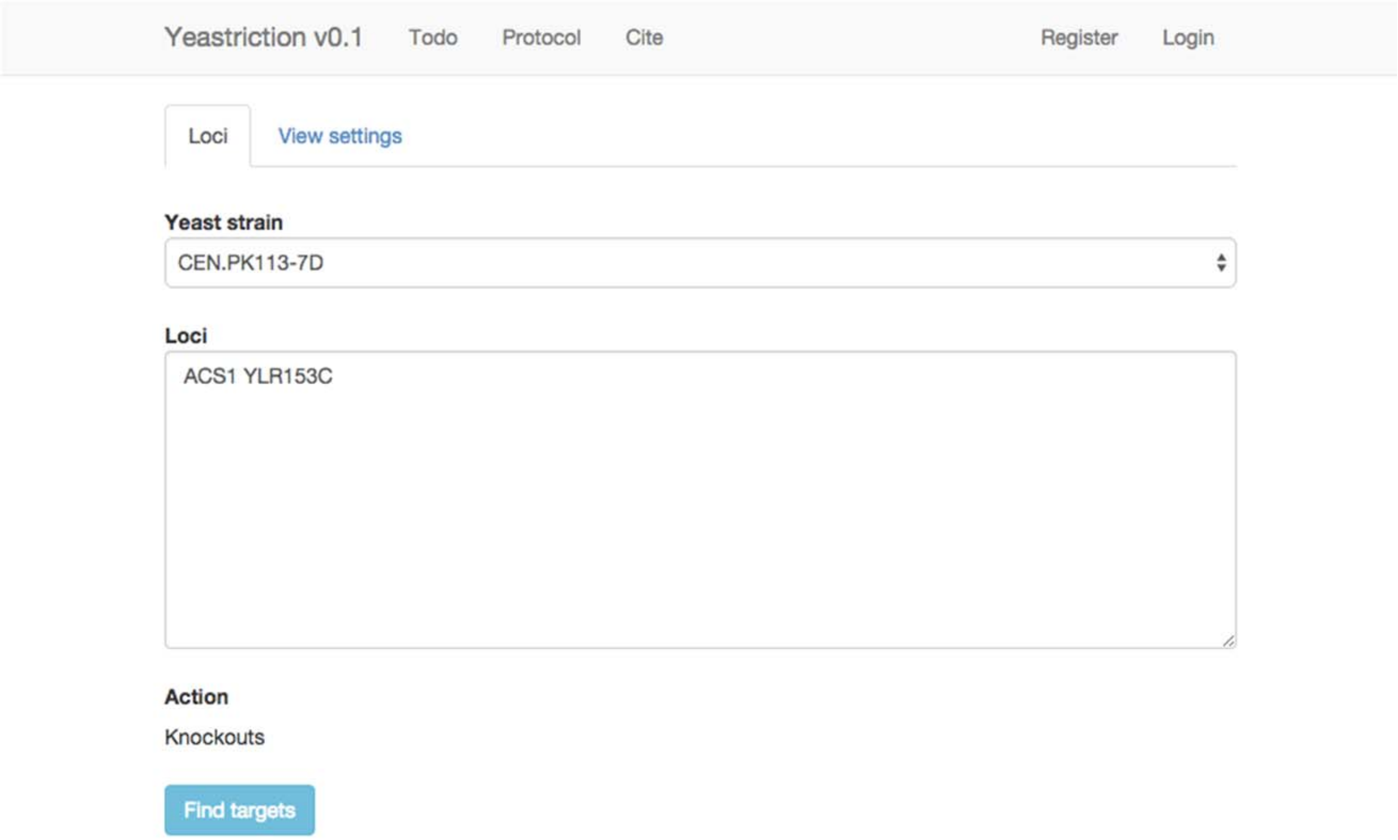

Click on Find targets.

Now the tool will give you the following screen.

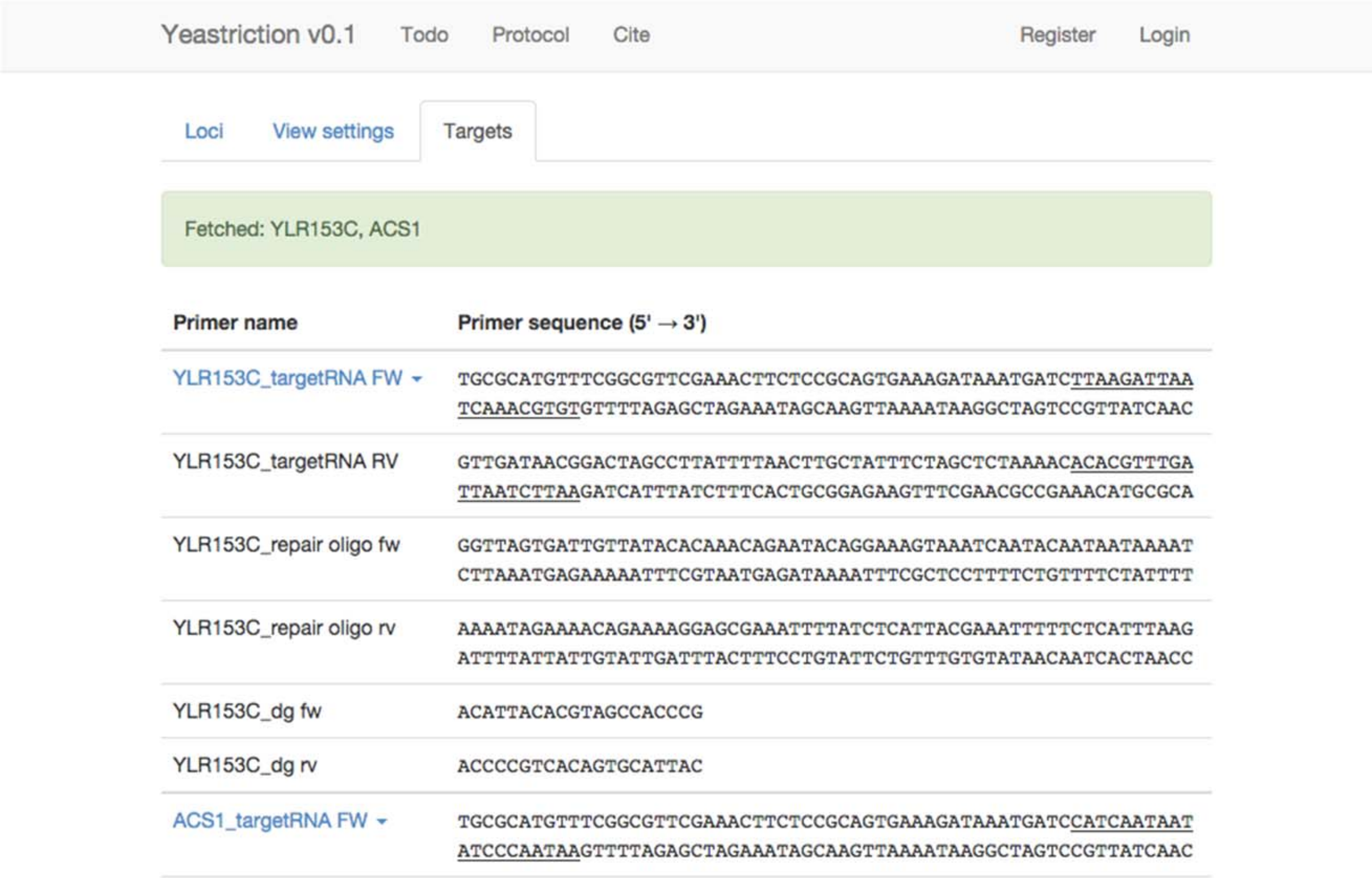

Primer name	Primer sequence (5' → 3')
YLR153C_targetRNA FW ▾	TGCGCATGTTTCGGCGTTCGAAACTTCTCCGCAGTGAAAGATAAATGATCTTAAGATTAATCAAACGTGTGTTTTAGAGCTAGAAATAGCAAGTTAAAATAAGGCTAGTCCGTTATCAAC
YLR153C_targetRNA RV	GTTGATAACGGACTAGCCTTATTTTAACTTGCTATTTCTAGCTCTAAAACACACGTTTGATTAATCTTAAGATCATTTATCTTTCACTGCGGAGAAGTTTCGAACGCCGAAACATGCGCA
YLR153C_repair oligo fw	GGTTAGTGATTGTTATACACAAACAGAATACAGGAAAGTAAATCAATACAATAATAAAATCTTAAATGAGAAAAATTTCGTAATGAGATAAAATTTCGCTCCTTTTCTGTTTTCTATTTT
YLR153C_repair oligo rv	AAAATAGAAAACAGAAAAGGAGCGAAATTTTATCTCATTACGAAATTTTTCTCATTTAAGATTTTATTATTGTATTGATTTACTTTCCTGTATTCTGTTTGTGTATAACAATCACTAACC
YLR153C_dg fw	ACATTACACGTAGCCACCCG
YLR153C_dg rv	ACCCCGTCACAGTGCATTAC
ACS1_targetRNA FW ▾	TGCGCATGTTTCGGCGTTCGAAACTTCTCCGCAGTGAAAGATAAATGATCCATCAATAATATCCCAATAAGTTTTAGAGCTAGAAATAGCAAGTTAAAATAAGGCTAGTCCGTTATCAAC

You can select another target sequence by clicking on the dropdown symbol.
When you select another target sequence the oligonucleotides will automatically be updated. The first column displays the target sequence, the second any restriction sites that might be present in the target sequence, the next columns displays the AT content, RNA score (number of unpaired nucleotides in the first 20 nucleotides divided by 20), the cumulated score followed by a column displaying the RNA structure in the dot-bracket notation; dots are unpaired nucleotides, opening brackets are nucleotides paired with the corresponding closing bracket.

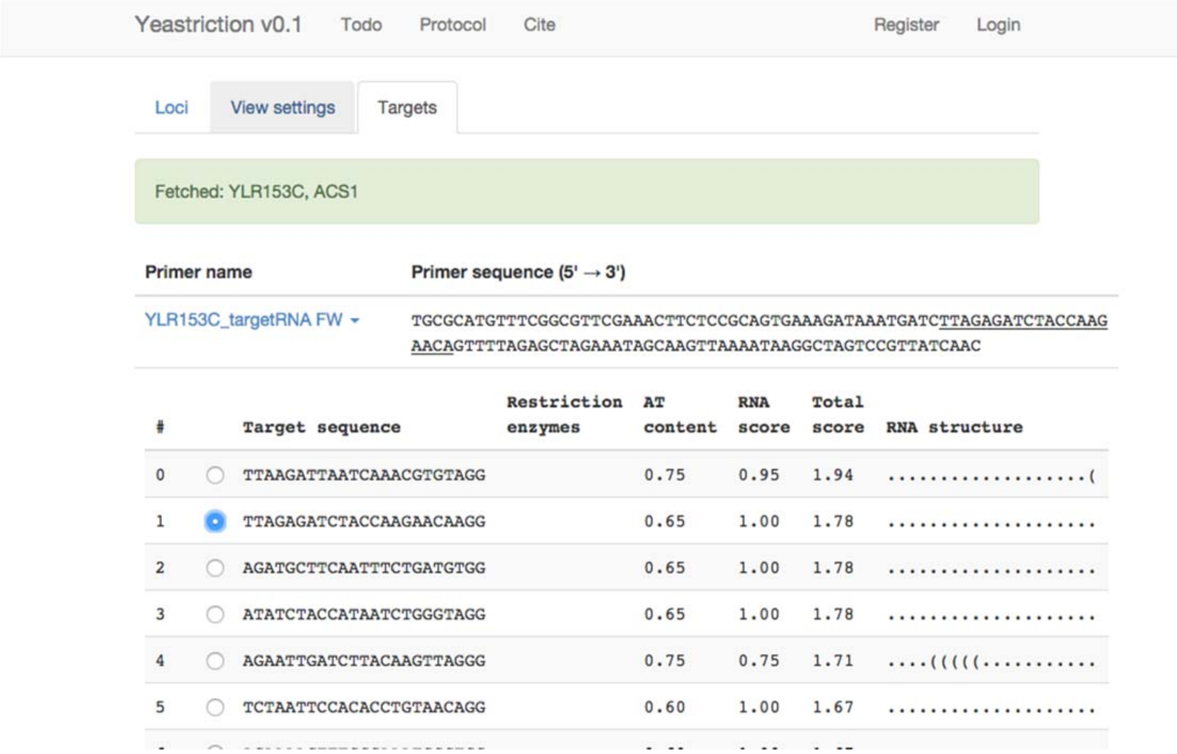

Primer name	Primer sequence (5' → 3')
YLR153C_targetRNA FW ▾	TGCGCATGTTTCGGCGTTCGAAACTTCTCCGCAGTGAAAGATAAATGATCTTAGAGATCTACCAAGAACAGTTTTAGAGCTAGAAATAGCAAGTTAAAATAAGGCTAGTCCGTTATCAAC

#		Target sequence	Restriction enzymes	AT content	RNA score	Total score	RNA structure
0	○	TTAAGATTAATCAAACGTGTAGG		0.75	0.95	1.94	...................(
1	◉	TTAGAGATCTACCAAGAACAAGG		0.65	1.00	1.78	....
2	○	AGATGCTTCAATTTCTGATGTGG		0.65	1.00	1.78	....
3	○	ATATCTACCATAATCTGGGTAGG		0.65	1.00	1.78	....
4	○	AGAATTGATCTTACAAGTTAGGG		0.75	0.75	1.71	....(((((...........
5	○	TCTAATTCCACACCTGTAACAGG		0.60	1.00	1.67	....
[illegible]	○	[illegible]		[illegible]	[illegible]	[illegible]	[illegible]

Next to the primer(s) incorporating the target sequences also oligonucleotides are given that can be used to repair the double strand break. The forward repair oligo is 60 bp upstream of the start codon concatenated with 60 bp of the sequence downstream of the stop codon. The reverse repair oligo is the reverse complement of this sequence. Also diagnostic primers are given to confirm gene deletion.

Under the tab "View settings" you can change same parameters, as shown in the following screenshot (the oligonucleotides are automatically updated).

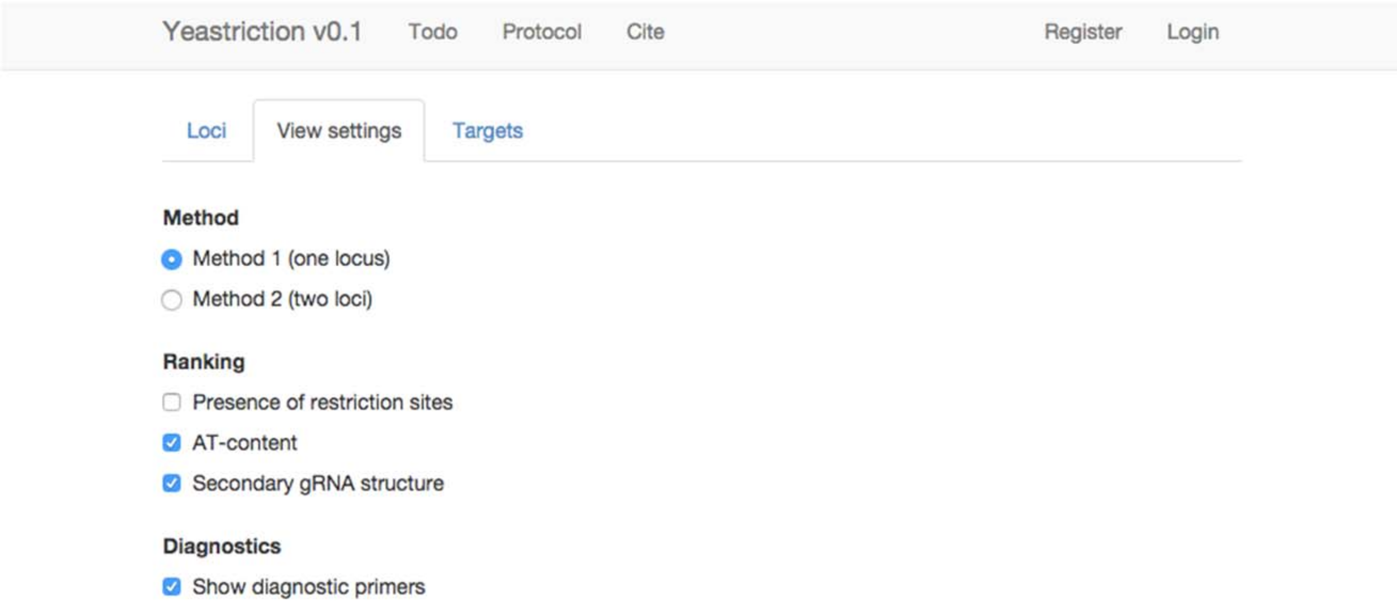

Protocol for gene editing using *in vivo* assembled plasmids containing single gRNAs

Introduction

The *in vivo* recombination method can be used to introduce marker- and scarless genetic modifications, is highly efficient for the creation of single modifications and has the advantage of a very simple workflow prior to yeast transformation. When aiming for multiple simultaneous genetic modifications, the *in vitro* recombination method is recommended. This protocol describes preparation of the DNA required for transformation (step 1 to 4), transformation into yeast (step 5), confirmation of the correct genotype (step 6) and removal of the guideRNA plasmid (step 7).

Steps

1. Design and order the guideRNA (gRNA) insert primers
    a. Design the gRNA primer using the Yeastriction tool at http://yeastriction.tnw.tudelft.nl.
    b. Alternatively, use the following complementary sequences as gRNA insert primers
    TGCGCATGTTTCGGCGTTCGAAACTTCTCCGCAGTGAAAGATAAATGATCN_{20}GTTTTAGAGCTAGAAATAGCAAGTTAAAATAAG
    GTTGATAACGGACTAGCCTTATTTTAACTTGCTATTTCTAGCTCTAAAACN_{c20}GATCATTTATCTTTCACTGCGGAGAAGTTTCGAACGCCGAAACATGCGCA
    (where N_{20} and N_{c20} are the complementary target sequences without the PAM sequence).
    c. Order the primers PAGE purified.
2. Construction of the double stranded gRNA insert
    a. Dissolve the complementary gRNA insert primers in distilled water to a final concentration of 100 µM.
    b. Mix the complementary primers in a 1:1 volume ratio.
    c. Heat the mixture to 95 °C for 5 min and subsequently anneal both primers by cooling down to room temperature on the bench.
3. Construction of the linearized backbone
    a. Prepare a PCR reaction mixture as follows:

Component	Amount (µL)	Final concentration
HF buffer	10	
dNTPs	1	200 µM
Phusion polymerase	0.5	0.02 U/µL
pMEL template*	1	1-5 ng/µL
Primer 6005	1	0.2 µM
Primer 6006	1	0.2 µM
Distilled water	35.5	
Total	50	

* this can be any pMEL plasmid

    b. Use the following conditions for the PCR

Step	Temperature (°C)	Time	
1	98	30 s	
2	98	10 s	
3	67	20 s	35 - 40x
4	68	3 min	
5	68	5 min	
6	12	∞	

c. After the PCR is finished, restrict the PCR mixture with FastDigest DpnI (Thermo Scientific) for 30 minutes according to the manufacturer's manual.
d. After digestion, load the complete reaction mixture on a 1% agarose 1xTAE gel with Serva staining.
e. Excise the PCR product and purify using the ZymoClean Gel DNA recovery kit according to manufacturer's instructions.

4. Construction of dsDNA repair fragment
   a. When aiming for a markerless deletion
      i. Use the Yeastriction tool at http://yeastriction.tnw.tudelft.nl.
      ii. Alternatively, design complementary 120 bp primers comprised of 60 bp sequences homologous to up- and downstream regions of the chromosomal target site, the sequence omitted between the up- and downstream regions will be removed from the chromosome during transformation.
      iii. Order the primers DST purified.
      iv. Anneal both primers as described in step 2.
   b. When aiming for mutations
      i. Design complementary 120 bp primers homologous to the 120 bp sequence around the chromosomal target site, introducing the desired mutation(s) in the 20 bp gRNA recognition sequence.
      ii. Order the primers PAGE purified.
      iii. Anneal both primers as described in step 2.
   c. When aiming for an integration
      i. Design primers for amplification of the desired DNA fragment for integration and add 60 bp 5' tails homologous to up- and downstream regions of the chromosomal target site, the sequence omitted between the up- and downstream regions will be replaced by the integration fragment during transformation.
      ii. Order the primers PAGE purified.
      iii. PCR amplify the insert fragment using Phusion polymerase.
      iv. After the PCR is finished, load the complete reaction mixture on a 1% agarose 1xTAE gel with Serva staining.
      v. Excise the PCR product and purify using the ZymoClean Gel DNA recovery kit according to manufacturer's instructions.
5. Yeast transformation
   a. Prepare the following transformation mix:

**Component**	**Amount**
Linearized backbone	100 ng
dsgRNA insert	300 ng
dsDNA repair fragment	0.2 - 1 μg
PEG	240 μL
1 M LiAc	36 μL
ssDNA	25 μL
Distilled water	X μL
Total	351 μL

   b. Transform *S. cerevisiae* with this mixture, according to the protocol of Gietz and Woods (Gietz *et al.*, 2002).

6. Confirmation of correct genetic modification
    a. DNA isolation (Lõoke *et al.*, 2011)
        i. Pick a yeast colony into 100 µL 0.2 M lithium acetate containing 1% SDS.
        ii. Alternatively, spin down 100 µL of a grown yeast culture in liquid medium, remove supernatant and resuspended in 100 µL 0.2 M lithium acetate containing 1% SDS.
        iii. Heat to 75 °C for 10 min.
        iv. Add 300 µL 100% ethanol and vortex.
        v. Spin down at maximum speed for 3 minutes and remove supernatant.
        vi. Resuspend the pellet in 150 µL 70% ethanol.
        vii. Spin down at maximum speed for 3 minutes and remove supernatant.
        viii. Dry the pellet at 37 °C, leaving the tube open.
        ix. Add 50-100 µL distilled water and vortex thoroughly.
        x. Spin down at maximum speed for 1 minute.
        xi. Use the supernatant directly as template for PCR.
    b. Design and order verification primers
        i. Using the Yeastriction tool at http://yeastriction.tnw.tudelft.nl.
        ii. Alternatively, design 20-30 bp forward and reverse primers, of which the resulting PCR product covers relevant homologous recombination sites to confirm correct DNA integration events during transformation.
        iii. Order the primers DST purified.
    c. Prepare a PCR reaction mixture as follows:

Component	Amount (µL)	Final concentration
DreamTaq MasterMix	10	
Template DNA (step 6a)	1-9	
Forward primer	0.5	0.25 µM
Reverse primer	0.5	0.25 µM
Distilled water		
Total	20	

    d. Use the following conditions for the PCR:

Step	Temperature (°C)	Time		
1	95	4	min	
2	95	30	s	
3	55	30	s	40x
4	72	1	min/kb	
5	72	10	min	
6	12	∞		

7. Plasmid removal
    a. Inoculate the confirmed culture in 25 mL of non-selective liquid medium
        i. When using auxotrophic markers this can be achieved by growing the cells on rich medium such as yeast peptone (YP) medium or via addition of the appropriate nutrients to synthetic medium and if possible compounds resulting in counterselection.
        ii. When using dominant markers this can be achieved by simply omitting or replacing the components associated with the dominant marker from the medium.
    b. Incubate the culture at 30 °C until the exponential growth phase is finished.
        i. The time required to achieve depletion of the carbon source heavily varies based on the strain background and medium composition.
    c. Streak part of the culture on a non-selective agar plates and incubate at 30 °C until single colonies are clearly visible.

d. Re-steak the obtained single colonies on non-selective plates and selective plates to confirm removal of the gRNA plasmid.
e. Transfer colonies that grow on non-selective, but not on selective medium agar plates to 20 mL non-selective liquid medium.
f. After sufficient cell growth, the culture can be stocked and stored at -80 °C and used for another round of transformation

Protocol for multiplexed gene editing using *in vitro* assembled plasmids containing two gRNAs

Introduction

Using this protocol, first a plasmid is constructed containing two guideRNAs facilitating restriction at two loci (step 1 to 5). After confirmation of the plasmid (step 6), it is transformed into yeast (step 7 to 8) with the appropriate repair fragments (step 2), following confirmation of the correct genotype (step 9) and removal of the guideRNA plasmid (step 10).

Steps

1. Design and order the guideRNA (gRNA) primers
    a. Design the gRNA primer using the Yeastriction tool at http://yeastriction.tnw.tudelft.nl.
    b. Alternatively, use the following sequence as gRNA primer
    `TGCGCATGTTTCGGCGTTCGAAACTTCTCCGCAGTGAAAGATAAATGATC`N_{20}`GTTTTAGAGCTAGAAATAGCAAGTTAAAATAAG`
    (where N_{20} is the target sequence without the PAM sequence, choosing a target site that contains a restriction site facilitates confirmation by restriction enzymes later on).
    c. Order the primer PAGE purified.
2. Construction of dsDNA repair fragment
    a. When aiming for markerless deletions
        i. Use the Yeastriction tool at http://yeastriction.tnw.tudelft.nl.
        ii. Alternatively, design complementary 120 bp primers comprised of 60 bp sequences homologous to up- and downstream regions of the chromosomal target site, the sequence omitted between the up- and downstream regions will be removed from the chromosome during transformation.
        iii. Order the primers DST purified.
        iv. Dissolve the complementary primers in distilled water to a final concentration of 100µM.
        v. Mix the complementary primers in a 1:1 volume ratio.
        vi. Heat the mixture to 95 °C for 5 min and subsequently anneal both primers by cooling down to room temperature on the bench.
    b. When aiming for mutations
        i. Design complementary 120 bp primers homologous to the 120 bp sequence around the chromosomal target site, introducing the desired mutation(s) in the 20 bp gRNA recognition sequence.
        ii. Order the primers PAGE purified.
        iii. Anneal both primers as described in step 2a.
    c. When aiming for an integration
        i. Design primers for amplification of the desired DNA fragment for integration and add 60 bp 5' tails homologous to up- and downstream regions of the chromosomal target site, the sequence omitted between the up- and downstream regions will be replaced by the integration fragment during transformation.
        ii. Order the primers PAGE purified.
        iii. PCR amplify the insert fragment using Phusion polymerase.
        iv. After the PCR is finished, load the complete reaction mixture on a 1% agarose 1xTAE gel with Serva staining.

v. Excise the PCR product and purify using the ZymoClean Gel DNA recovery kit according to manufacturer's instructions.

3. Construction of the 2µm fragment
   a. Set-up a PCR reaction for production of the 2µm fragment as follows:
   (We use DreamTaq (left column) and Phusion (right column) interchangeably)

DreamTaq

**Component**	**Amount (µL)**
DreamTaq MasterMix	50
pROS template*	80 ng
gRNA primer 1¥	2
gRNA primer 2¥	2
Distilled water	X
Total volume	100

Phusion

**Component**	**Amount (µL)**	**Final concentration**
HF buffer	20	
dNTPs	2	200 µM
Phusion	1.5	0.03 U/µL
pROS template*	80 ng	
gRNA primer 1¥	2	0.2 µM
gRNA primer 2¥	2	0.2 µM
Distilled water	X	
Total volume	100	

* this can be any pROS plasmid

¥ as design in step 1a or 1b

   b. Divide the 100 µL reaction mixture in two PCR tubes.
   c. Use the following conditions for the PCR:

DreamTaq

**Step**	**Temperature (°C)**	**Time**		
1	95	4	min	
2	95	30	s	
3	55	30	s	40x
4	66	2	min	
5	66	10	min	
6	12	∞		

Phusion

**Step**	**Temperature (°C)**	**Time**		
1	98	3	min	
2	98	30	s	
3	69	30	s	40x
4	72	3	min	
5	72	10	min	
6	12	∞		

   d. After the PCR is finished, load the complete reaction mixture on a 1% agarose 1xTAE gel with Serva staining.
   e. Excise the 1589 bp PCR product on a Safe Imager™ 2.0 Blue-Light Transilluminator (Life Technologies) and purify using the ZymoClean Gel DNA recovery kit (Zymoclean™, D2004, Zymo Research, Irvine, CA) according to manufacturer's instructions.

4. Construction of the linearized backbone
   a. Prepare a PCR reaction mixture as follows:

**Component**	**Amount (µL)**	**Final concentration**
HF buffer	10	
dNTPs	1	200 µM
Phusion polymerase	0.75	0.03 U/µL
pROS template*	80 ng	
primer 6005	2	0.4 µM
Distilled water	X	
Total volume	50	

* this can be any pROS plasmid

   b. Use the following conditions for the PCR

**Step**	**Temperature (°C)**	**Time**		
1	98	3	min	
2	98	30	s	
3	63	30	s	35-40x
4	68	2	min	

5	68	10 min
6	12	∞

c. After the PCR is finished, restrict the PCR mixture with FastDigest DpnI (Thermo Scientific) for 30 minutes according to the manufacturer's manual.
d. After digestion, load the complete reaction mixture on a 1% agarose 1xTAE gel with Serva staining.
e. Excise the PCR product and purify using the ZymoClean Gel DNA recovery kit according to manufacturer's instructions.

5. Assembly the 2μm fragment with the backbone using Gibson Assembly
   a. Prepare the following Gibson Assembly reaction mixture:

**Component**	**Amount**
Gibson Assembly MasterMix*	5 μL
2μm fragment	100 ng
backbone	100 ng
Distilled water	X μL
Total	10 μL

   * New England Biolabs
   b. Incubate the reaction mixture at 50 °C for 1 hour in a thermocycler.
   c. After the reaction is finished, add 20 μL of distilled water and transform 2 μL to DH5α *E. coli* using electroporation in a 2 mm cuvette (165-2086, BioRad, Hercules, CA) with a Gene PulserXcell Electroporation System (BioRad), following the manufacturer's protocol.
   d. Plate *E. coli* cells on LB with 100 mg·L^{-1} ampicillin
   e. After incubating ~18 h at 37 °C pick ~4-6 colonies and transfer to 15 mL BD Falcon tubes containing 4 mL of liquid LB with 100 mg·L^{-1} ampicillin
   f. Incubate ~18 h at 37 °C in a shaker
6. Confirmation of constructed plasmid
   a. Extract the plasmids from 2 mL of *E. coli* culture using the Sigma GenElute Plasmid kit (Sigma-Aldrich), according to the supplier's manual
   (after confirmation, the remaining 2 mL may be used for stocking)
   b. Elute the plasmid with 60 μL of buffer instead of 100 μL
   c. Confirm the plasmid with either restriction analysis or diagnostic PCR
      i. Confirmation using restriction analysis
         a. If the target sequence contains a restriction site, this may be used to confirm the plasmid.
         b. Design restriction experiment
         ▲ Be aware that there are 4 different PCR products of the 2μm fragment:
         1. [target_sequence_1]-[2μm]-[target_sequence_1]
         2. [target_sequence_1]-[2μm]-[target_sequence_2]
         3. [target_sequence_2]-[2μm]-[target_sequence_1]
         4. [target_sequence_2]-[2μm]-[target_sequence_2]
         Moreover, the 2μm fragment can be oriented in two directions. So there are 8 different possible plasmids, of which 4 are of the desired architecture.
         c. Use the following recipe:

**Component**	**Amount**
FastDigest Green Buffer*	2 μL
Plasmid	500 ng
FastDigest Enzyme(s)*	1 μL
Distilled water	X μL
Total	20 μL

         * ThermoScientific

d. After incubating for ~30 minutes at the temperature specified by the manufacturer, load the entire reaction mix on a 1% agarose 1x TAE gel with Serva staining.
e. Confirm correct plasmid assembly by visualizing bands using the UV transillumination G-Box (Syngene)

ii. Alternatively, use PCR to confirm correct plasmid assembly
a. Design primers that are complementary to the target sequence (see Figure 1, primer α and primer β, for, respectively, target sequence 1 and 2)
b. For every plasmid use the following two PCR reactions:

	**Component**	**Amount (μL)**	**Final concentration**
**Reaction 1**	DreamTaq MasterMix	10	
	plasmid	3	
	Primer α	0.5	0.25 μM
	Primer A*	0.5	0.25 μM
	Primer B*	0.5	0.25 μM
	Distilled water	x	

* For the sequences of primer A and B see Table 1. These primers can be used for confirmation of any pROS plasmid.
And

	**Component**	**Amount (μL)**	**Final concentration**
**Reaction 2**	DreamTaq MasterMix	10	
	plasmid	3	
	Primer β	0.5	0.25 μM
	Primer A	0.5	0.25 μM
	Primer B	0.5	0.25 μM
	Distilled water	x	

c. Run the PCR, following this procedure:

**Step**	**Temperature (°C)**	**Time**	
1	95	4 min	
2	95	30 s	
3	55	30 s	35x
4	72	30 s	
5	72	10 min	
6	12	∞	

d. Load the PCR reaction mixture on a 1% agarose 1xTAE gel with Serva staining
e. Confirm correct plasmid assembly by visualizing bands using UV transillumination equipment
▲ If the plasmid shows the architecture as shown in figure 1A, one band at 379 bp is expected for reaction 1, and at 552 bp for reaction 2. If the situation is as in Figure 1B, the fragment for reaction 1 should be 552 bp and for reaction 2 379 bp. If the plasmid is as in Figure 1C, two fragments are expected with reaction 1, at 379 bp and 552 bp (and no bands for reaction 2). If the plasmid is as Figure 1D, no bands are expected for reaction 1 and two bands for reaction 2, at 379 bp and 552 bp.

7. Inoculate the correct clone to 20 mL LB with 100 mg·L^{-1} ampicillin in a 50 mL erlenmeyer, ensuring sufficient aeration
a. Spin down the 20 mL of culture in Falcon tubes.

b. Resuspend the cell pellet with 800 µL of resuspension buffer and divide in two 1.5 mL reaction tubes.
c. Follow the rest of the Sigma GenElute Plasmid kit (Sigma-Aldrich) protocol, with the following modifications:
   i. Adjust the volume of lysis buffer and neutralization buffer to 400 µL and 700 µL, respectively.
   ii. Per reaction tube, load the reaction mixture in two steps of 750 µL to a column
   iii. Elute with 30 µL

8. Yeast transformation
   a. Prepare the dsDNA repair fragment as in step 2.
   b. Prepare the following transformation mix:

Component	Amount
Plasmid	2 µg
dsDNA repair fragment	1 - 2.5 µg
PEG	240 µL
1 M LiAc	36 µL
ssDNA	25 µL
Distilled water	X µL
Total	351 µL

   c. Transform *S. cerevisiae* with this mixture, according to the protocol of Gietz and Woods (Gietz *et al.*, 2002).

9. Confirmation of correct genetic modification
   a. DNA isolation (Lõoke *et al.*, 2011)
      i. Pick a yeast colony into 100 µL 0.2 M lithium acetate containing 1% SDS.
      ii. Alternatively, spin down 100 µL of a grown yeast culture in liquid medium, remove supernatant and resuspend in 100 µL 0.2 M lithium acetate containing 1% SDS.
      iii. Heat to 75°C for 10 min.
      iv. Add 300 µL 100% ethanol and vortex.
      v. Spin down at maximum speed for 3 minutes and remove supernatant.
      vi. Resuspend the pellet in 150 µL 70% ethanol.
      vii. Spin down at maximum speed for 3 minutes and remove supernatant.
      viii. Dry the pellet at 37 °C, leaving the tube open.
      ix. Add 50-100 µL distilled water and vortex thoroughly.
      x. Spin down at maximum speed for 1 minute.
      xi. Use the supernatant directly as template for PCR.
   b. Design and order verification primers
      i. Using the Yeastriction tool at http://yeastriction.tnw.tudelft.nl.
      ii. Alternatively, design 20-30 bp forward and reverse primers, of which the resulting PCR product covers relevant homologous recombination sites to confirm correct DNA integration events during transformation.
      iii. Order the primers desalted.
   c. Prepare a PCR reaction mixture as follows:

Component	Amount (µL)	Final concentration
DreamTaq MasterMix	10	
Template DNA (step 6a)	1-9	
Forward primer	0.5	0.25 µM
Reverse primer	0.5	0.25 µM
Distilled water		
Total	20	

   d. Use the following conditions for the PCR:

Step	Temperature (°C)	Time

1	95	4 min	
2	95	30 s	
3	55	30 s	40x
4	72	1 min/kb	
5	72	10 min	
6	12	∞	

10. Plasmid removal
    a. Inoculate the confirmed culture in 25 mL of non-selective liquid medium
        i. When using auxotrophic markers this can be achieved by growing the cells on rich medium such as yeast peptone (YP) medium or via addition of the appropriate nutrients to synthetic medium.
        ii. When using dominant markers this can be achieved by simply omitting or replacing the components associated with the dominant marker from the medium.
    b. Incubate the culture at 30 °C until the exponential growth phase is finished.
        i. The time required to achieve depletion of the carbon source heavily varies based on the strain background and medium composition.
    c. Steak part of the culture on a non-selective agar plates and incubate at 30 °C until single colonies are clearly visible.
    d. Re-steak the obtained single colonies on non-selective plates and selective plates to confirm removal of the gRNA plasmid.
    e. Transfer colonies that grow on non-selective, but not on selective medium agar plates to 20 mL non-selective liquid medium.
    f. After sufficient cell growth, the culture can be stocked and stored at -80 °C and used for another round of transformation.

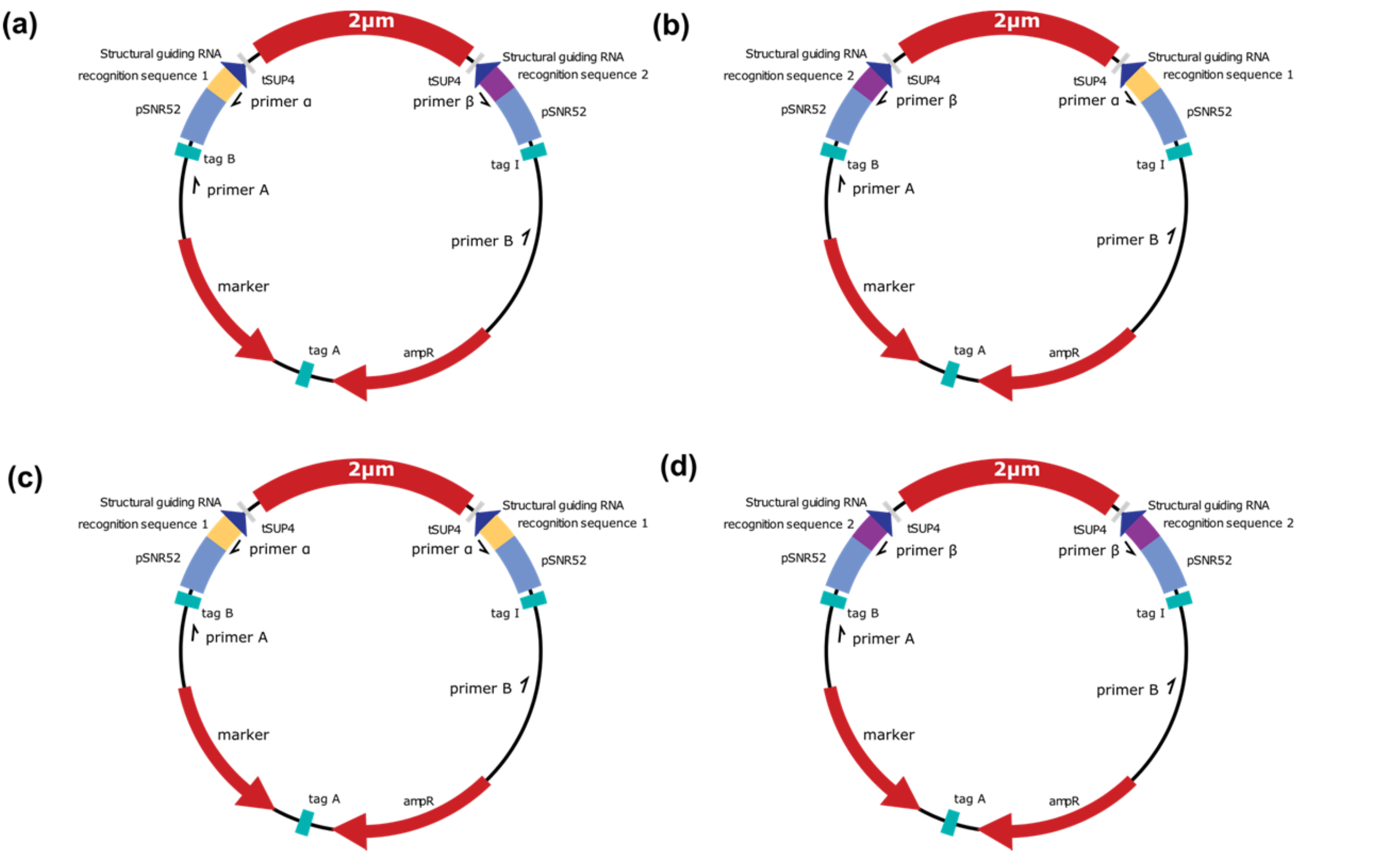

Figure 1
When using the double gRNA approach to assemble the fragment [target_sequence_1 or 2]-[2µm]-[target_sequence_1 or 2] with backbone there are eight possible end products. **(a)** [target_sequence_1]-[2µm]-[target_sequence_2] **(b)** [target_sequence_2]-[2µm]-[target_sequence_1] **(c)** [target_sequence_1]-[2µm]-[target_sequence_1] **(d)** [target_sequence_ 2]-[2µm]-[target_sequence_

2]. The four other possible assemblies are the same to A – D, except that the 2μm fragment is in reverse.

Table 1

Name	Sequence 5' → 3'
Primer A	CACCTTTCGAGAGGACGATG
Primer B	GCTGGCCTTTTGCTCACATG

References

Gietz DR & Woods RA (2002) Transformation of yeast by lithium acetate/single-stranded carrier DNA/polyethylene glycol method. *Methods Enzymol* **350**: 87–96.

Lõoke M, Kristjuhan K & Kristjuhan A (2011) Extraction of genomic DNA from yeasts for PCR-based applications. *Biotechniques* **50**: 325–328.

Supplementary Table S1

Primers used in this study

Number	Name	Sequence 5' → 3'
***CAS9* integration**		
2873	CAN1DelcassFW	TCAGACTTCTTAACTCCTGTAAAAACAAAAAAAAAAAAAGGCATAGCAATAAGCTGGAGCTCATAGCTTC
3093	tagA-pUG	ACTATATGTGAAGGCATGGCTATGGCACGGCAGACATTCCGCCAGATCATCAATAGGCACCTTCGTACGCTGCAGGTCGAC
4653	A-CYC1t-rv	GTGCCTATTGATGATCTGGCGGAATGTCTGCCGTGCCATAGCCATGCCTTCACATATAGTCCGCAAATTAAAGCCTTCGAG
5542	CAN1 KO rv	CTATGCTACAACATTCCAAAATTTGTCCCAAAAAGTCTTTGGTTCATGATCTTCCCATACGCATAGGCCACTAGTGGATCTG
**Cas9 integration confirmation**		
5829	CAN1 cut rv	AGAAGAGTGGTTGCGAACAGAG
2673	m-PCR-HR4-RV	TGAAGTGGTACGGCGATGC
2668	m-PCR-HR2-FW	ACGCGTGTACGCATGTAAC
9	KanA	CGCACGTCAAGACTGTCAAG
2620	Nat Ctrl Fw	GCCGAGCAAATGCCTGCAAATC
2615	Can1RV	GAAATGGCGTGGGAATGTGA
**Construction pMEL series**		
3093	tagA-pUG	ACTATATGTGAAGGCATGGCTATGGCACGGCAGACATTCCGCCAGATCATCAATAGGCACCTTCGTACGCTGCAGGTCGAC
3096	tagB-pUG	GTTGAACATTCTTAGGCTGGTCGAATCATTTAGACACGGGCATCGTCCTCTCGAAAGGTGGCATAGGCCACTAGTGGATCTG
6845	p426 cRNA-rv A	GTGCCTATTGATGATCTGGCGGAATGTCTGCCGTGCCATAGCCATGCCTTCACATATAGTACAGGCAACACGCAGATATAGG
6846	p426 cRNA-fw B	CACCTTTCGAGAGGACGATGCCCGTGTCTAAATGATTCGACCAGCCTAAGAATGTTCAACGGCCCACTACGTGAACCATC
6847	pRS Marker fw A	ACTATATGTGAAGGCATGGCTATGGCACGGCAGACATTCCGCCAGATCATCAATAGGCACTGCGGCATCAGAGCAGATTG
6848	pRS Marker rv B	GTTGAACATTCTTAGGCTGGTCGAATCATTTAGACACGGGCATCGTCCTCTCGAAAGGTGCATCTGTGCGGTATTTCACACC
6005	p426 CRISPR rv	GATCATTTATCTTTCACTGCGGAGAAG
6006	p426 CRISPR fw	GTTTTAGAGCTAGAAATAGCAAGTTAAAATAAGGCTAGTC
**Construction pROS series**		
5972	tSUP4 rv ol 2mu-5*	GTTCTACAAAATGAAGCACAGATGCTTCGTTGGAGGGCGTGAACGTAAG
5974	2mu inside fw	TACTTTTGAGCAATGTTTGTGGA
5975	2mu inside rv	AACGAGCTACTAAAATATTGCGAA
6007	struct-guideRNA ADE2.y (ol pSNR52)	GTGCGCATGTTTCGGCGTTCGAAACTTCTCCGCAGTGAAAGATAAATGATCACTTGAAGATTCTTTAGTGTGTTTTAGAGCTAGAAATAGCAAGTTAAAATAAG
6008	struct-guideRNA CAN1.y (ol pSNR52)	GTGCGCATGTTTCGGCGTTCGAAACTTCTCCGCAGTGAAAGATAAATGATCGATACGTTCTCTATGGAGGAGTTTTAGAGCTAGAAATAGCAAGTTAAAATAAG
3289	Fus Tag F fw	CATACGTTGAAACTACGGCAAAGG
4692	Fus Tag G rv	AAGGGCCATGACCACCTG
5976	pSNR52 fw ol tag I-2	GCCTACGGTTCCCGAAGTATGCTGCTGATGTCTGGCTATACCTATCCGTCTACGTGAATACCCTCACTAAAGGGAACAAAAG
5977	pSNR52 fw ol tag B	CACCTTTCGAGAGGACGATGCCCGTGTCTAAATGATTCGACCAGCCTAAGAATGTTCAACCCCTCACTAAAGGGAACAAAAG
5973	tSUP4 rv ol 2mu 3*	GGCATAGTGCGTGTTTATGCTTAAATGCGTGGAGGGCGTGAACGTAAG
5975	2mu inside rv	AACGAGCTACTAAAATATTGCGAA
4068	Nic1 amp Fwd	GCCTACGGTTCCCGAAGTATGC
5974	2mu inside fw	TACTTTTGAGCAATGTTTGTGGA
3841	for f3h	CACCTTTCGAGAGGACGATG
3847	FUS Tag A fw	ACTATATGTGAAGGCATGGCTATGG
3276	Fus Tag B-rv	GTTGAACATTCTTAGGCTGGTCGAATC
3274	Fus Tag I-fw	TATTCACGTAGACGGATAGGTATAGC
3275	Fus Tag A-rv	GTGCCTATTGATGATCTGGCGGAATG
5793	pCAS9 rv	GATCATTTATCTTTCACTGCGGAG
**Construction PDH cassettes**		
5654	D_FW_E1a	GAATTCACGCATCTACGACTG
7426	tTEF1_yeast ol tACS2	GAAAATAGAAAACAGAAAAGGAGCGAAATTTTATCTCATTACGAAATTTTTCTCATTTAAGGAGGCACTATTACTGATGTGATTTC
3277	Fus Tag C rv	CTAGCGTGTCCTCGCATAGTTCTTAGATTG
7338	pTDH3 fw ol tag I	TATTCACGTAGACGGATAGGTATAGCCAGACATCAGCAGCATACTTCGGGA

		ACCGTAGGCATAAAAAACACGCTTTTTCAGTTCG
3284	Fus Tag J rv	CGACGAGATGCTCAGACTATGTGTTC
7356	tPGI1 ol pACS2	GGTTAGTGATTGTTATACACAAACAGAATACAGGAAAGTAAATCAATACAA<br>TAATAAAATTAATTTTTAAAATTTTTACTTTTCGCGAC
5653	D_RV_E3	AAGCTTAATCACTCTCCATACAGGG
3283	Fus Tag C fw	ACGTCTCACGGATCGTATATGC
5661	I_RV_LL.LA1	TCTAGAGCCTACGGTTCCCGA
5663	H_FW_LA1	TCTAGAAGATTACTCTAACGCCTCAGC
2686	Tag H fusion reverse	GTCACGGGTTCTCAGCAATTCG
3285	Fus Tag J fw	GGCCGTCATATACGCGAAGATGTC
**gRNA cassette construction**		
6835	CrRNA insert MCH1 XhoI FW	TGCGCATGTTTCGGCGTTCGAAACTTCTCCGCAGTGAAAGATAAATGATCT<br>ATTGGCAATAAACATCTCGGTTTTAGAGCTAGAAATAGCAAGTTAAAATAA<br>GGCTAGTCCGTTATCAAC
6836	CrRNA insert MCH1 XhoI RV	GTTGATAACGGACTAGCCTTATTTTAACTTGCTATTTCTAGCTCTAAAACC<br>GAGATGTTTATTGCCAATAGATCATTTATCTTTCACTGCGGAGAAGTTTCG<br>AACGCCGAAACATGCGCA
6837	CrRNA insert MCH2 PvuI FW	TGCGCATGTTTCGGCGTTCGAAACTTCTCCGCAGTGAAAGATAAATGATCA<br>TCTCGATCGAGGTGCCTGAGTTTTAGAGCTAGAAATAGCAAGTTAAAATAA<br>GGCTAGTCCGTTATCAAC
6838	CrRNA insert MCH2 PvuI RV	GTTGATAACGGACTAGCCTTATTTTAACTTGCTATTTCTAGCTCTAAAACT<br>CAGGCACCTCGATCGAGATGATCATTTATCTTTCACTGCGGAGAAGTTTCG<br>AACGCCGAAACATGCGCA
6839	CrRNA insert MCH5 EcoRV FW	TGCGCATGTTTCGGCGTTCGAAACTTCTCCGCAGTGAAAGATAAATGATCA<br>CTCTTCCGTTTTAGATATCGTTTTAGAGCTAGAAATAGCAAGTTAAAATAA<br>GGCTAGTCCGTTATCAAC
6840	CrRNA insert MCH5 EcoRV RV	GTTGATAACGGACTAGCCTTATTTTAACTTGCTATTTCTAGCTCTAAAACG<br>ATATCTAAAACGGAAGAGTGATCATTTATCTTTCACTGCGGAGAAGTTTCG<br>AACGCCGAAACATGCGCA
6841	CrRNA insert AQY1 DraI FW	TGCGCATGTTTCGGCGTTCGAAACTTCTCCGCAGTGAAAGATAAATGATCA<br>CCATCGCTTTAAAATCTCTGTTTTAGAGCTAGAAATAGCAAGTTAAAATAA<br>GGCTAGTCCGTTATCAAC
6842	CrRNA insert AQY1 DraI RV	GTTGATAACGGACTAGCCTTATTTTAACTTGCTATTTCTAGCTCTAAAACA<br>GAGATTTTAAAGCGATGGTGATCATTTATCTTTCACTGCGGAGAAGTTTCG<br>AACGCCGAAACATGCGCA
6843	CrRNA insert ITRI EcoRI FW	TGCGCATGTTTCGGCGTTCGAAACTTCTCCGCAGTGAAAGATAAATGATCA<br>TACATCAACGAATTCCAACGTTTTAGAGCTAGAAATAGCAAGTTAAAATAA<br>GGCTAGTCCGTTATCAAC
6844	CrRNA insert ITRI EcoRI RV	GTTGATAACGGACTAGCCTTATTTTAACTTGCTATTTCTAGCTCTAAAACG<br>TTGGAATTCGTTGATGTATGATCATTTATCTTTCACTGCGGAGAAGTTTCG<br>AACGCCGAAACATGCGCA
7040	CrRNA insert PDR12 KpnI FW	TGCGCATGTTTCGGCGTTCGAAACTTCTCCGCAGTGAAAGATAAATGATCG<br>CATTTTCGGTACCTAACTCGTTTTAGAGCTAGAAATAGCAAGTTAAAATAA<br>GGCTAGTCCGTTATCAAC
7041	CrRNA insert PDR12 KpnI RV	GTTGATAACGGACTAGCCTTATTTTAACTTGCTATTTCTAGCTCTAAAACG<br>AGTTAGGTACCGAAAATGCGATCATTTATCTTTCACTGCGGAGAAGTTTCG<br>AACGCCGAAACATGCGCA
7348	ACS2_targetRNA FW	TGCGCATGTTTCGGCGTTCGAAACTTCTCCGCAGTGAAAGATAAATGATCT<br>CCTTGCCGTTAAATCACCAGTTTTAGAGCTAGAAATAGCAAGTTAAAATAA<br>GGCTAGTCCGTTATCAAC
6414	ACS1 gRNA	GTGCGCATGTTTCGGCGTTCGAAACTTCTCCGCAGTGAAAGATAAATGATC<br>TTCTTCACAGCTGGAGACATGTTTTAGAGCTAGAAATAGCAAGTTAAAATA<br>AG
7026	GET4_targetRNA FW BamHI, DpnI	TGCGCATGTTTCGGCGTTCGAAACTTCTCCGCAGTGAAAGATAAATGATCG<br>GGCTCGCTAGGATCCAATTGTTTTAGAGCTAGAAATAGCAAGTTAAAATAA<br>GGCTAGTCCGTTATCAAC
7032	NAT1_targetRNA FW BamHI, DpnI	TGCGCATGTTTCGGCGTTCGAAACTTCTCCGCAGTGAAAGATAAATGATCA<br>AAGGAATTGGATCCTGCGTGTTTTAGAGCTAGAAATAGCAAGTTAAAATAA<br>GGCTAGTCCGTTATCAAC
**Repair oligos**		
6849	MCH1 Repair Oligo FW	GGTGTCATTTATATAAGCTATGAATTTTAAAAAAAATAAATGTAGCAGTTT<br>CTTTTTTGTGATTTGCACTTGGAAAATGGTTATTGCTATAAAATGATATGA<br>AAGGAAACTAGTCTCGAT
6850	MCH1 Repair Oligo RV	ATCGAGACTAGTTTCCTTTCATATCATTTTATAGCAATAACCATTTTCCAA<br>GTGCAAATCACAAAAAAGAAACTGCTACATTTATTTTTTTTAAAATTCATA<br>GCTTATATAAATGACACC
6851	MCH2 Repair Oligo FW	TATAGAACTATATAACTGATACTAGAATATACTAATTCGTGCACTATTAAC<br>CGTTTGGCGAGGTCACTTTTATTTCACACTGTAGATAAGAAGGGGATAGAG<br>TTGCCAGAAAATTTTTTG
6852	MCH2 Repair Oligo RV	CAAAAAATTTTCTGGCAACTCTATCCCCTTCTTATCTACAGTGTGAAATAA<br>AAGTGACCTCGCCAAACGGTTAATAGTGCACGAATTAGTATATTCTAGTAT

		CAGTTATATAGTTCTATA
6853	MCH5 Repair Oligo FW	TAAAAGAAAATATTATTGCATTACTTTTTTGAAGATCTATAAAGGGCACTGTCTTACTTTTATTTTTCTTTTTAATCTATAGTAAAATCAGAGCTTTTTAATCGATAGTATGCCCCCGTG
6854	MCH5 Repair Oligo RV	CACGGGGGCATACTATCGATTAAAAAGCTCTGATTTTACTATAGATTAAAAAGAAAAATAAAAGTAAGACAGTGCCCTTTATAGATCTTCAAAAAAGTAATGCAATAATATTTTCTTTTA
6855	AQY1 Repair Oligo FW	CTTTGTATTTGGTGCTGTCTGTCAATACGGCACATAAAGTAACATGTAATTAACTATAACTTTTTCCCTCCTTTTCTTTATTTCTCGCTCACTAGCACTTAATGTTATAATACTCGGCAA
6856	AQY1 Repair Oligo RV	TTGCCGAGTATTATAACATTAAGTGCTAGTGAGCGAGAAATAAAGAAAAGGAGGGAAAAAGTTATAGTTAATTACATGTTACTTTATGTGCCGTATTGACAGACAGCACCAAATACAAAG
6857	ITR1 Repair Oligo FW	ATTTTCTACTATGTATTTGAATATTCAATTGCGTCTCCTTCCTTTTACCTCGTGAAAGGATTTAACACCCACTGCAGAAACAAAGAAAATGAAAGAGATGTATACAGTAGGACGACCAAT
6858	ITR1 Repair Oligo RV	ATTGGTCGTCCTACTGTATACATCTCTTTCATTTTCTTTGTTTCTGCAGTGGGTGTTAAATCCTTTCACGAGGTAAAAGGAAGGAGACGCAATTGAATATTCAAATACATAGTAGAAAAT
7042	PDR12 Repair Oligo FW	AAAATTGAAAATAAAAATTGTGTGTTAAACCACGAAATACAAATATATTTGCTTGCTTGTTTTTTTATTAATAAGAACAATAACAATAAATCTGTAAACCTTTTTTTTAAGTGAAAATTA
7043	PDR12 Repair Oligo RV	TAATTTTCACTTAAAAAAAAGGTTTACAGATTTATTGTTATTGTTCTTATTAATAAAAAAACAAGCAAGCAAATATATTTGTATTTCGTGGTTTAACACACAATTTTTATTTTCAATTTT
6422	ACS1 repair fw	CTATCTATAAGCAAAACCAAACATATCAAAACTACTAGAAAGACATTGCCCCACTGTGTTTGATGATTTCTTTCCTTTTTATATTGACGACTTTTTTTTTCGTGTGTTTTTGTTCTCTTA
6423	ACS1 repair rv	TAAGAGAACAAAAACACACGAAAAAAAAAGTCGTCAATATAAAAAGGAAAGAAATCATCAAACACAGTGGGGCAATGTCTTTCTAGTAGTTTTGATATGTTTGGTTTTGCTTATAGATAG
7028	GET4_repair oligo fw	GGAAGTTAAAGTTGATGACATCTCAGTTGCTAGATTGGTTAGATTAATAGCCGAATTCGATCCTAGCGAGCCCAATTTAAAGGACGTTATTACTGGTATGAACAATTGGTCTATCAAATT
7029	GET4_repair oligo rv	AATTTGATAGACCAATTGTTCATACCAGTAATAACGTCCTTTAAATTGGGCTCGCTAGGATCGAATTCGGCTATTAATCTAACCAATCTAGCAACTGAGATGTCATCAACTTTAACTTCC
7034	NAT1_repair oligo fw	GTTTCACCACTATTGGAGAAAATTGTCCTTGATTATTTGTCCGGATTAGATCCTACGCAGGATCGAATTCCTTTTATTTGGACCAATTATTACTTGTCTCAACATTTCCTTTTCCTTAAG
7035	NAT1_repair oligo rv	CTTAAGGAAAAGGAAATGTTGAGACAAGTAATAATTGGTCCAAATAAAAGGAATTCGATCCTGCGTAGGATCTAATCCGGACAAATAATCAAGGACAATTTTCTCCAATAGTGGTGAAAC
**Diagnostic primers for confirming correct double gRNA plasmid assembly**		
Primer A		CACCTTTCGAGAGGACGATG
Primer B		GCTGGCCTTTTGCTCACATG
7351	GET4 dg fw-2	AATTGGATCCTAGCGAGCCC
7352	NAT1 dg fw-2	ACGCAGGATCCAATTCCTTTG
**Diagnostic primers for seamless deletion confirmation**		
6862	MCH1 check FW	GTCCAGGATTCTCCGAAGAACTC
6863	MCH1 check RV	ACGGCTGTTTGTCCGATATTGC
6864	MCH2 check FW	GCACGACTTTGCAGGCTTTC
6865	MCH2 check RV	CACCGAACCAACATTAGGTAGC
6866	MCH5 check FW	GAAGACTGACGGGCACTTTG
6867	MCH5 check RV	GCCCTAGGCGGTATTGTATGAG
6868	AQY1 check FW	GATGTCCTTCCACCCTCTTACAC
5593	42 - CBS1483_Sc16_Scf39 _HPA2-Rv	CGTGTATTGGTGTACGGATGAGTC
6869	ITR1 check FW	AGGTCTTCAATGCCGGGTTAG
6870	ITR1 check RV	GTATCAGCCGTCAGTAGTATCCAG
253	PDR12 - CTRL FW	CGGTATCACATTTTCTCGACGG
3998	PDR12 KO rv	CGCGACAGACATTTGTTGG
**Diagnostic primers for *GET4* and *NAT1* mutation confirmation**		
7030	GET4_dg fw	TGTTCGTTTTGTCGCTCTTCG
7031	GET4_dg rv	AACTCAGCCACCGTGCTATC
7036	NAT1_dg fw	TATCCAAGATGCGAACCACCC
7037	NAT1_dg rv	AGATAGGCTCTTGGCGTACC
**Diagnostic primers for confirming strain IMX719**		
2619	acs2 KO Ctrl Rv	CCGATATTCGGTAGCCGATTCC
2430	Tag C-reverse short	GCGTCCAAGTAACTACATTATGTG
2668	m-PCR-HR2-FW	ACGCGTGTACGCATGTAAC
7330	NATDiagfw	CGAGCAAATGCCTGCAAATC

5044	ADH1 Flank right FW	CTAGCGGTTATGCGCGTCTCAC
2913	G_RV_2 (PDH construct ctrl)	AATAGCCGCCAGGAAATGCC
2914	J_FW (PDH construct ctrl)	GTCGTCATAACGATGAGGTGTTGC
2915	J_RV (PDH construct ctrl)	GGAGCCAACAAGAATAAGCCGC
2908	D_FW (PDH construct ctrl)	GGATTGGGTGTGATGTAAGGATTCGC
2909	D_RV (PDH construct ctrl)	CCCGCTCACACTAACGTAGG
2916	I_FW (PDH construct ctrl)	GCAGGTATGCGATAGTTCCTCAC
2905	B_RV (PDH construct ctrl)	TGGCAGTATTGATAATGATAAACTCG
5641	F_FW_EF	CCATTGCTGAAGCTGACAAG
2618	acs2 Ctrl Fw	TACCCTATCCCGGGCGAAGAAC
2927	ACS1 KO ctrl FW	AAACTGGGCGGCTATTCTAAGC
2928	ACS1 KO ctrl RV	AGCAGCTCGGTTATAAGAGAAC

Supplementary data S1

Alignment of 120 bp surrounding the target site at the mutated *GET4* locus of the four sequenced colonies (in bold the target site, in lowercase the intended mutation, in yellow additional, undesired mutations).

**Reference**	258	GGAAGTTAAAGTTGATGACATCTCAGTTGCTAGATTGGTTAGATTAATAG**CCGAATTGGA**
Colony #1		GGAAGTTAAAGTTGATGACATCTCAGTTGCTAGATTGGTTAGATTAATAGCCGAATTcGA
Colony #2		GGAAGTTAAAGTTGATGACATCTCAGTTGCTAGATTGGTTAGATTAATAGCCGAATTcGA
Colony #4		GGAAGTTAAAGTTGATGACATCTCAGTTGCTAGATTGGTTAGATTAATAGCCGAATTcGA
Colony #6		GGAAGTTAAAGTTGATGACATCTCAGTTGCTAGATTGGTTAGATTAATAGCCGAATTcGA

**Reference**	318	**TCCTAGCGAGCCC**AATTTAAAGGACGTTATTACTGGTATGAACAATTGGTCTATCAAATT
Colony #1		TCCTAGCGAGCCCAATTTAAAGGACGTTATTACTGGTATGAACAATTGGTCTATCAAATT
Colony #2		TCCTAGCGAGCCCAATTTAAAG-------TTACTGGTATGAACAATTGGTCTATCAAATT
Colony #4		T-CTAGCGAGCCCAATTTAAAGGACGTTATTATTGGTATGAACAATTGGTCTATCAAATT
Colony #6		TCCTAGCGAGCCCAATTTAAAGGACGTTATTACTGGTATGAACAATTGGTCTATCAAATT

Alignment of 120 bp surrounding the target site at the mutated *NAT1* locus of the four sequenced colonies (in bold the target site, in lowercase the intended mutation).

**Reference**	1075	GTTTCACCACTATTGGAGAAAATTGTCCTTGATTATTTGTCCGGATTAGAT**CCTACGCAG**
Colony #1		GTTTCACCACTATTGGAGAAAATTGTCCTTGATTATTTGTCCGGATTAGATCCTACGCAG
Colony #2		GTTTCACCACTATTGGAGAAAATTGTCCTTGATTATTTGTCCGGATTAGATCCTACGCAG
Colony #4		GTTTCACCACTATTGGAGAAAATTGTCCTTGATTATTTGTCCGGATTAGATCCTACGCAG
Colony #6		GTTTCACCACTATTGGAGAAAATTGTCCTTGATTATTTGTCCGGATTAGATCCTACGCAG

**Reference**	1135	**GATCCAATTCCTTT**TATTTGGACCAATTATTACTTGTCTCAACATTTCCTTTTCCTTAAG
Colony #1		GATCgAATTCCTTTTATTTGGACCAATTATTACTTGTCTCAACATTTCCTTTTCCTTAAG
Colony #2		GATCgAATTCCTTTTATTTGGACCAATTATTACTTGTCTCAACATTTCCTTTTCCTTAAG
Colony #4		GATCgAATTCCTTTTATTTGGACCAATTATTACTTGTCTCAACATTTCCTTTTCCTTAAG
Colony #6		GATCgAATTCCTTTTATTTGGACCAATTATTACTTGTCTCAACATTTCCTTTTCCTTAAG
